# On the reversibility of parasitism: adaptation to a free-living lifestyle via gene acquisitions in the diplomonad *Trepomonas* sp. PC1

**DOI:** 10.1186/s12915-016-0284-z

**Published:** 2016-08-01

**Authors:** Feifei Xu, Jon Jerlström-Hultqvist, Martin Kolisko, Alastair G. B. Simpson, Andrew J. Roger, Staffan G. Svärd, Jan O. Andersson

**Affiliations:** 1 Department of Cell and Molecular Biology, Science for Life Laboratory, Uppsala University, Uppsala, Sweden; 2 Department of Biology, Dalhousie University, Halifax, NS Canada; 3 Department of Biochemistry and Molecular Biology, Dalhousie University, Halifax, NS Canada; 4 Canadian Institute for Advanced Research, Integrated Microbial Biodiversity Program, Toronto, ON Canada; 5 Present address: Department of Medical Biochemistry and Microbiology, Uppsala University, Uppsala, Sweden; 6 Present address: Botany Department, University of British Columbia, Vancouver, BC Canada

**Keywords:** Free-living, Parasite, Diplomonad, Dollo’s law, Reversibility, *Trepomonas*, Horizontal gene transfer, Ribonucleotide reductase

## Abstract

**Background:**

It is generally thought that the evolutionary transition to parasitism is irreversible because it is associated with the loss of functions needed for a free-living lifestyle. Nevertheless, free-living taxa are sometimes nested within parasite clades in phylogenetic trees, which could indicate that they are secondarily free-living. Herein, we test this hypothesis by studying the genomic basis for evolutionary transitions between lifestyles in diplomonads, a group of anaerobic eukaryotes. Most described diplomonads are intestinal parasites or commensals of various animals, but there are also free-living diplomonads found in oxygen-poor environments such as marine and freshwater sediments. All these nest well within groups of parasitic diplomonads in phylogenetic trees, suggesting that they could be secondarily free-living.

**Results:**

We present a transcriptome study of *Trepomonas* sp. PC1, a diplomonad isolated from marine sediment. Analysis of the metabolic genes revealed a number of proteins involved in degradation of the bacterial membrane and cell wall, as well as an extended set of enzymes involved in carbohydrate degradation and nucleotide metabolism. Phylogenetic analyses showed that most of the differences in metabolic capacity between free-living *Trepomonas* and the parasitic diplomonads are due to recent acquisitions of bacterial genes via gene transfer. Interestingly, one of the acquired genes encodes a ribonucleotide reductase, which frees *Trepomonas* from the need to scavenge deoxyribonucleosides. The transcriptome included a gene encoding squalene-tetrahymanol cyclase. This enzyme synthesizes the sterol substitute tetrahymanol in the absence of oxygen, potentially allowing *Trepomonas* to thrive under anaerobic conditions as a free-living bacterivore, without depending on sterols from other eukaryotes.

**Conclusions:**

Our findings are consistent with the phylogenetic evidence that the last common ancestor of diplomonads was dependent on a host and that *Trepomonas* has adapted secondarily to a free-living lifestyle. We believe that similar studies of other groups where free-living taxa are nested within parasites could reveal more examples of secondarily free-living eukaryotes.

**Electronic supplementary material:**

The online version of this article (doi:10.1186/s12915-016-0284-z) contains supplementary material, which is available to authorized users.

## Background

The word parasite originates from Greek *parasitos* meaning “a person who eats at the table of another”. In biology, the word is used for a relationship where an organism (the parasite) uses resources of another organism (the host), and lives on or inside that organism. The historical view of parasites is that they are simplified versions of free-living organisms. This view is, however, outdated, as it has become increasingly clear that parasites are organisms highly adapted to their specific niches [1]. The transition from a free-living to a parasitic lifestyle is an evolutionary process that includes the loss of some existing functions as well as the gain of new functions needed to survive on or within the host, transmit between hosts and exploit the resources from the host [2]. It is often argued that this evolutionary transition from a free-living state to parasitism is irreversible. The rationale is that parasites take advantage of resources from the host, leading to specialized and reductive evolution including, often, a simplified metabolism [2–6]. Once such dependence has evolved, it would seem to be nearly impossible to revert to a more complex metabolism as was found in free-living ancestors. This has sometimes been taken as an example of Dollo’s law, which states that a complex trait cannot re-evolve in the same form [7, 8].

The idea of irreversibility of parasitism is widespread; in an overview of 15 parasitology books, only four mentioned reversals to a free-living state as a possible, but unlikely, evolutionary path [8]. However, this paradigm in biology has been questioned [8]. Free-living house dust mites and certain nematodes have been proposed to have evolved from parasitic ancestors [9, 10]. There is strong phylogenetic support for house dust mites being secondarily free-living [10], but the genetics behind this lifestyle transition remains unknown. Diplomonads are another group in which free-living members may have evolved from host-associated ancestors, based on phylogenetic analyses [1, 8, 11]. Herein, we examine the hypothesis of a parasitic ancestry for free-living diplomonads using a transcriptome sequencing approach, with the aim of revealing the genomic basis and evolutionary origins of the lifestyle differences within the group.

**Fig. 1:**
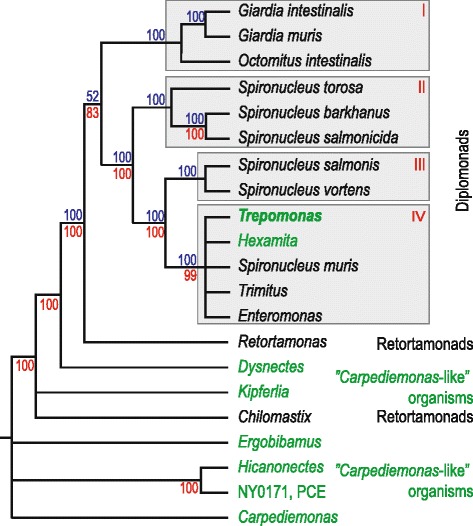
Phylogeny of diplomonads and their closest relatives. The schematic tree is based on a combination of two published phylogenetic analyses [22, 23]. Maximum likelihood bootstrap support values from a ribosomal RNA analysis [22] and a multigene analysis [23] are shown in blue and red, respectively. Support values are missing from some branches because the two analyses contained only partly overlapping taxa: species outside retortamonads are missing in the ribosomal RNA study [22] and some of the diplomonad species are missing in the multigene study [23]. Taxa shown in green are predominantly or entirely free-living. *Chilomastix* and *Trimitus* are predominantly host-associated, but include some free-living isolates

Diplomonads are a group of flagellated protists belonging to the taxon Excavata [12, 13]. Their closest relatives within the group are Retortamonads, *Carpediemonas*, and a range of poorly-studied lineages collectively known as *Carpediemonas*-like organisms (Fig. 1). Most diplomonads have a characteristic ‘double karyomastigont’; the presence of two identical nuclei and two flagellar apparatuses per cell [12]. Diplomonads are characteristically found in oxygen-poor environments such as sediments and the intestinal tract of animals [12]. Most described diplomonads are associated with various hosts as parasites or commensals. The best studied is *Giardia intestinalis*, an enteric parasite that infects a wide range of animals [14]. In humans, *G. intestinalis* cause diarrhea and other symptoms. The prevalence of *Giardia* in humans is high in some regions, and there are hundreds of millions of infections per year worldwide [15]. *Spironucleus salmonicida* was the first diplomonad outside of *Giardia* to be studied on the genome level. *S. salmonicida*, previously known as *Spironucleus barkhanus* [16], is an intestinal parasite of fish that can also cause systemic infection, invading the blood stream and different organs of its host [17, 18].

*G. intestinalis* and *S. salmonicida* share adaptations to a parasitic lifestyle such as a large family of cysteine-rich proteins that likely provide antigenic variation to escape the host immune system and an encystation pathway that enables them to transmit between hosts [19, 20]. They are dependent on scavenging of metabolites from the host, exemplified by the absence of a ribonucleotide reductase (RNR) needed for synthesis of deoxyribonucleotides [21]. These similarities indicate that they could share a common parasitic ancestor. Comparison between *G. intestinalis* and *S. salmonicida* also revealed differences. For example, the latter has an extended metabolic repertoire with more potential for gene regulation. This difference is likely a result of their different habitats, as *Giardia* lives in a stable gut environment, while *S. salmonicida* is adapted to a fluctuating environment [20].

The diplomonads studied with molecular methods can be tentatively divided into four monophyletic groups based on molecular data (Fig. 1) [22, 23]. Three of the groups (I–III) only contain taxa described as parasites or commensals (Fig. 1). Group I includes *G. intestinalis*, a parasite adapted to the environment of the intestine of humans and other mammals [19, 24, 25]. Groups II and III contain members of the genus *Spironucleus*, which are mostly dependent on fish for their survival as parasites or commensals [26]. *S. vortens* is the causative agent of hole-in-the-head disease in ornamental fish [27, 28], *S. barkhanus*, *S. salmonicida* and *S. salmonis* are associated with wild and farmed salmonid fish [16, 18, 29–31], and *S. torosa* is associated with gadidae [32]. Group IV contains both diplomonads found in association with host species, such as *S. muris* [33], and free-living diplomonads, for example (most representatives of) the genera *Trepomonas* and *Hexamita* [12]. The fact that free-living diplomonads are exclusively found in group IV, nested within host-associated lineages with strong statistical support (Fig. 1), has invited two possible explanations: (1) that diplomonads have adapted to life within a host several times independently, or (2) that the free-living lifestyle of diplomonads such as *Trepomonas* and *Hexamita* is a secondary adaptation from a host-associated ancestor [11, 34]. If diplomonads were ancestrally free-living, we expect genomic features associated with this lifestyle, such as enzymes for degradation of bacterial prey, to be shared with free-living eukaryotes. If, on the other hand, *Trepomonas* recently adapted to life outside an animal host, these features should have evolved after the divergence from the lineages leading to *G. intestinalis* and *S. salmonicida*. We could annotate almost 8000 gene fragments in the *Trepomonas* transcriptome and among these found hundreds of genes recently acquired from various sources. Many of the laterally transferred genes are associated with degradation of phagocytosed bacteria, an important trait for a free-living heterotrophic protist.

## Results

### Identification of *Trepomonas* sp. PC1 genes in a transcriptome of a mixed culture

We assembled 41 million single reads into 18,527 transcripts with length ≥ 200 nt using Inchworm [35]; 9980 genes were called from the transcripts and annotated based on the *S. salmonicida* genome [20], supplemented with information from protein and domain databases. The annotation algorithm is described in detail in the Method section. The *Trepomonas* culture was monoeukaryotic, but included a mixture of bacteria. Thus, mRNA extracted for sequencing was expected to contain bacterial contamination, even though the RNA was polyA-purified before library construction.

The fact that hexamitine diplomonads (groups II–IV in Fig. 1) use a non-canonical genetic code [36, 37] greatly aided the detection of contamination in the dataset. *Trepomonas* utilizes the codons TAA and TAG to encode glutamine instead of termination, leaving TGA as the only stop codon. This alternative genetic code has been identified in a few other eukaryotic lineages (e.g., some ciliates [38]), but not, to our knowledge, in any prokaryote. We excluded from further analyses a total of 1995 fragments of genes as possible contaminants because they had higher similarity to non-eukaryotic sequences than to any eukaryotic sequence and contained less than two in-frame TAA/TAG codons.

We identified 7985 *Trepomonas* gene fragments, 6106 of which have matches in sequence or domain databases (Table 1). The vast majority (96 %) of the annotated sequences have at least two in-frame TAA/TAG codons, showing that they are true *Trepomonas* genes (Table 1). Most of the remaining 4 % are likely to be *Trepomonas* genes that lack TAA/TAG codons by chance; three quarters of these actually have their best matches to *S. salmonicida* genes. There could still be bacterial transcripts within this dataset if our culture were contaminated with a yet-to-be identified lineage utilizing a genetic code identical to diplomonads. However, this is very unlikely because we did not find any bacterial ribosomal proteins in the *Trepomonas* dataset, while there were 114 such proteins lacking in-frame TAA/TAG codons among the transcripts excluded as contamination. The details on how contaminating sequences were removed are given in the Methods section. The coding regions have an average GC content of 39.0 %, which is similar to *S. salmonicida* [20]; 1692 genes have orthologs in the *S. salmonicida* genome, with an average level of amino acid identity of 42.1 % (Additional file 1: Figure S1).

**Table 1:** Details of the annotation

	With BLAST hit	With only Pfam hit	No hit	Total
Count	5910	196	1879	7985
# In-frame TAA/TAG in hit ≥ 2	5361	146	–	5507
# In-frame TAA/TAG ≥ 2	5592	185	1877	7654
Avg GC%	39.4	36.7	38.3	39.0

We used BUSCO [39] to estimate how complete the gene content is with the *Trepomonas* transcriptome; 143 of the 429 conserved eukaryotic proteins provided by BUSCO were identified within the *Trepomonas* transcriptome, compared to 138 and 173 for the genomes of *S. salmonicida* and *G. intestinalis* [19, 20], respectively. This suggests that the *Trepomonas* transcriptome dataset includes the majority of the protein-coding genes in the genome.

### *Trepomonas* sp. PC1 has an extended coding capacity compared to parasitic diplomonads

The metabolic capacity of the fish parasite *S. salmonicida* was previously found to be expanded compared to the human parasite *G. intestinalis* [20]. We here extend the analysis to include the free-living *Trepomonas* sp. PC1 (Table 2) and find that *Trepomonas* has more genes in most functional categories than either *G. intestinalis* or *S. salmonicida*. This further suggests that our transcriptome of *Trepomonas* represents most of the genes in the genome. The differences are in agreement with lifestyle differences. *Trepomonas* has most genes in eight of the 11 categories involving metabolism (Table 2), suggesting that the free-living diplomonad has a more elaborate metabolism capable of utilizing a wider range of metabolites. *Trepomonas* also has more genes involved in transport, signal transduction and cellular communication (Table 2), suggesting adaptation to a less stable environment than the intestine of salmonids (*S. salmonicida*) or mammals (*G. intestinalis*).

**Table 2:** Functional pathway differences identified in the KAAS analysis

	*Trepomonas* ^a^	*S. salmonicida* ^a^	*G. intestinalis* ^a^
Carbohydrate metabolism	60 (74)	52 (66)	33 (34)
Energy metabolism	35 (43)	36 (44)	27 (29)
Lipid metabolism	28 (44)	19 (24)	14 (16)
Nucleotide metabolism	62 (75)	48 (57)	47 (51)
Amino acid metabolism	33 (40)	29 (41)	15 (16)
Metabolism of other amino acids	15 (19)	12 (15)	6 (6)
Glycan biosynthesis and metabolism	8 (8)	8 (9)	11 (11)
Metabolism of cofactors and vitamins	28 (35)	23 (29)	15 (17)
Metabolism of terpenoids and polyketides	5 (6)	5 (5)	13 (13)
Biosynthesis of other secondary metabolites	8 (10)	3 (7)	2 (2)
Xenobiotics biodegradation and metabolism	15 (19)	9 (10)	7 (7)
Transport and catabolism	65 (93)	52 (71)	49 (60)
Transcription	35 (39)	29 (30)	52 (55)
Membrane transport	9 (9)	4 (4)	3 (3)
Signal transduction	77 (95)	62 (78)	58 (65)
Cellular communication	24 (28)	19 (28)	17 (23)

^a^Number of distinct KEGG Orthology and number of enzymes in parenthesis

Gene transfer of prokaryotic genes into eukaryotic lineages is a common mechanism for adaptation that is acting on different evolutionary timescales [40–42]. For example, analyses of the genome of the moss *Physcomitrella patens* suggested that gene transfer was important for the plant colonization of land [43], and a recent study showed that transfer of genes have occurred in historical times between fungi used in cheese making [44]. Gene transfer has been identified as an evolutionary mechanism affecting diplomonad genomes [19, 45]. Reported cases include ancient events shared with the parabasalid *Trichomonas vaginalis* [45], as well as an example of recent acquisition where an isolate of *G. intestinalis* encodes a functional bacterial gene flanked by two pseudogenes of bacterial origin [46].

We performed a phylogenomic analysis of all *Trepomonas* sp. PC1 transcripts to detect genes that have been gained relatively recently from non-eukaryotic sources. Genes unique to *Trepomonas* among sampled diplomonads would indicate probable cases of functions gained by the free-living diplomonad. We used a combination of the programs Phylogenie [47] and Darkhorse [48] to identify transfer candidates. RAxML trees were constructed for the candidates predicted by either of the programs, and were then inspected manually. We identified 423 transfer candidates that corresponded to 271 transfer events (Additional file 2: Table S1; Additional file 3). Among them there are 40 transfer events, corresponding to 61 genes, for which we could infer a putative donor lineage with bootstrap support ≥ 70 %. No dominating lineage could be identified among the putative donors, suggesting that these genes have been acquired in a large number of individual events. The majority of the transfers were from Bacteria, with only eight from Archaea and one from a virus. Among Bacteria, Proteobacteria and Firmicutes were the most common donor groups, with 12 and 10 cases, respectively. Functions associated with degradation of bacterial prey were abundant among the transfer candidates.

### General lysosomal degradation

Phagocytized prey needs to be digested to recover components for anabolic processes. Eukaryotes fuse their phagocytic vacuoles with lysosomes that deliver a cocktail of hydrolytic enzymes that degrade captured prey. We found many *Trepomonas* proteins that are potentially involved in this process, for example, lysosomal proteases (cathepsins), glycosidases (GBA, HexA/B), palmitoyl-thioesterases, lipases (LYPLA3), nucleases (DNaseII), saposins, hydrolases, and hemolysins. Several of these proteins have been transferred from bacterial sources where they perform these functions in the absence of a lysosome (Additional file 2: Table S1).

Bactericidal permeability-increasing proteins (BPI) have antimicrobial activity in humans [49]. These proteins bind to the lipid A moiety of lipopolysaccharides of the outer membrane of Gram-negative bacteria and penetrate into the inner membrane, thereby causing cell death. However, members of this protein family have diverse functional roles and very little is known about their function outside animals [50]. We detect 40 BPI-like proteins in *Trepomonas* and they are also present in multiple copies in the *S. salmonicida* and *Giardia* genomes (Additional file 4: Figure S2). The high sequence diversity and large number of BPI-like proteins in *Trepomonas* sp. PC1 suggest that the family is functionally diverse. Some of these proteins may have bactericidal functions, although biochemical studies are needed to understand the role of BPI-like proteins in various diplomonads.

Transglutaminases are enzymes that catalyze formation of isopeptide bonds, although some members of this family perform the reverse reaction [51]. The formation of isopeptide bonds may serve to entrap or clot bacteria to prevent escape from the phagosome or to establish contact at the interface between the eukaryotic microbe and a bacterium [52]. *Trepomonas* encodes a set of proteins with transglutaminase domains that have been laterally transferred from bacteria and which may serve these functions (Additional file 2: Table S1). Functional studies are needed to test whether these proteins are functional within a lysosome in *Trepomonas*.

### Degradation of bacterial cell walls

**Fig. 2:**
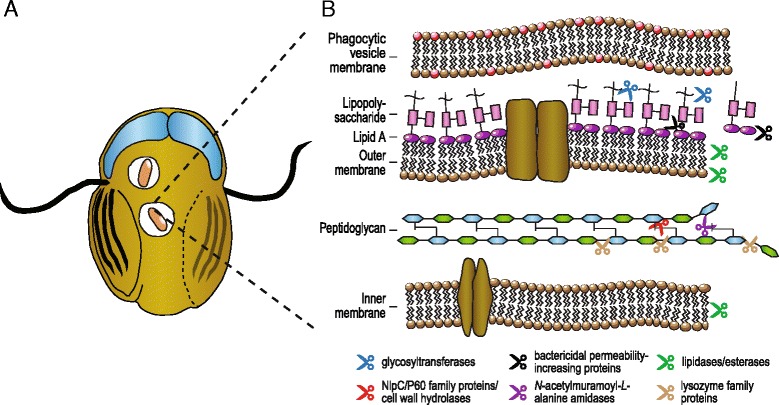
*Trepomonas* proteins potentially involved in phagocytosis and digestion of bacterial prey. **a** Illustration of a *Trepomonas* sp. PC1 cell with phagocytosed bacteria (*pink*), two nuclei (*blue*) and eight flagella, two of which are used for motility. **b** Proteins predicted to have hydrolytic activity acting on the Gram-negative bacterial cell wall and membranes are indicated with scissors: glycosyltransferases (*blue*), bactericidal permeability-increasing proteins (*black*), lipidases/esterases (*green*), NlpC/P60 family proteins or cell wall hydrolases (*red*), N-acetylmuramoyl-L-alanine amidases (*purple*), and lysozyme family proteins (*brown*)

The great majority of bacteria have peptidoglycan cell walls that protect the cell from lysis under environmental stressors. We identified a set of proteins within the *Trepomonas* transcriptome data that degrade different components of bacterial cell walls and likely enable the free-living diplomonad to feed on a variety of bacteria (Fig. 2).

**Fig. 3:**
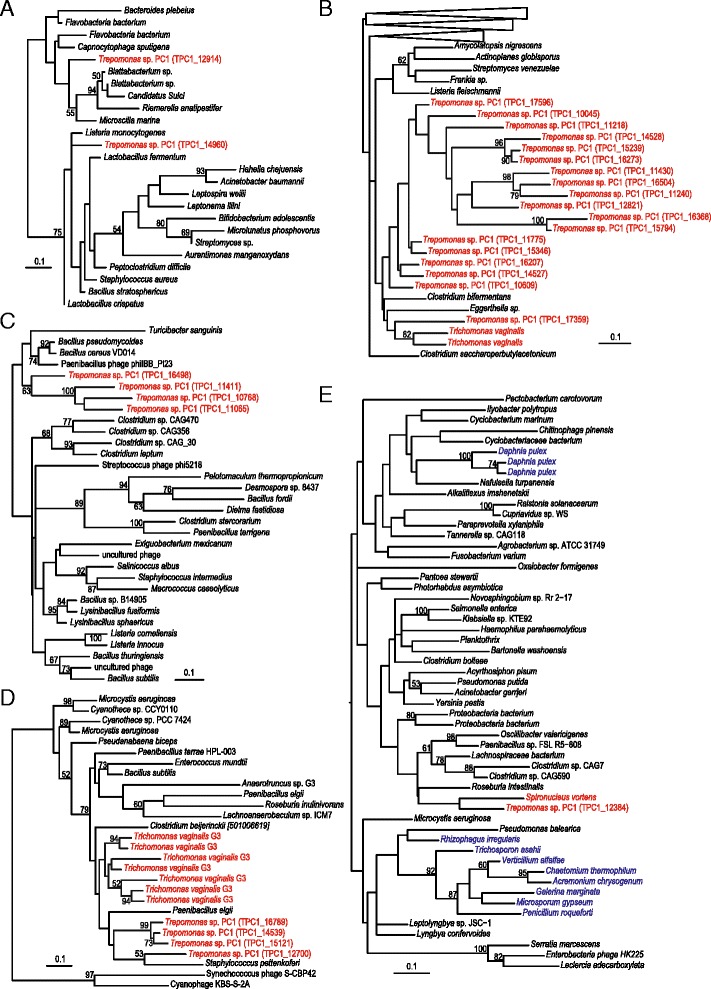
Maximum likelihood phylogenies of proteins involved in degradation of bacterial prey: **a** cell-wall hydrolase, **b** NlpC/P60 family protein, **c** N-acetylmuramoyl-L-alanine amidase, **d** lysozyme-family protein, and **e** lysozyme-family protein. Eukaryotes are labeled in color: Excavata (*red*), Opisthokonta (*blue*). Only bootstrap support values > 50 % are shown

Cell wall hydrolases are peptidases that target the isopeptide bonds of peptidoglycan, with most characterized members hydrolyzing D-γ-glutamyl-meso-diaminopimelate or *N*-acetylmuramate-*L*-alanine cross-links [53, 54] (Fig. 2). Cell wall hydrolases are absent from both *Spironucleus* and *Giardia* but *Trepomonas* sp. PC1 has some 20 homologs, with multiple phylogenetic origins (Fig. 3a, b). Two *Trepomonas* cell wall hydrolases branch separately within bacteria, suggestive of two independent origins, although the conserved part of the protein is short and, consequently, the phylogenetic tree is poorly resolved (Fig. 3a). The other 18 cell wall hydrolases contain the NlpC/P60 family domain and form two poorly supported groups in the phylogenetic analysis (Fig. 3b). The absence of homologs in other diplomonads suggests that at least one NlpC/P60 family protein gene was acquired by an ancestor of *Trepomonas* after the divergence from *Spironucleus* and subsequently expanded via gene duplications.

*Trepomonas* also encodes four bacterial *N*-acetylmuramoyl-*L*-alanine amidases (peptidoglycan amidases) that are predicted to degrade peptidoglycan cross-links at the stem peptide of Mur*N*Ac and *L*-alanine (Fig. 2). Peptidoglycan amidases are very common in bacteria, where they assist in the maintenance, elongation and division of the peptidoglycan sacculus, but are rarely found in eukaryotes [55, 56]. Mammals and some other animals encode homologs of peptidoglycan amidase (peptidoglycan recognition proteins) that bind to the cell wall and kill the bacterium by activating a protein-sensing two-component system [57]. The four *Trepomonas* peptidoglycan amidases are found in a single cluster in the phylogenetic tree, nested among bacterial and phage proteins (Fig. 3c), indicating that they have been acquired via gene transfer.

Lysozyme-family proteins cleave β-1,4-glycosidic bonds between the Mur*N*Ac and Glc*N*Ac groups in peptidoglycan (or between the Glc*N*Ac residues of chitin) and thereby cause lysis of bacterial cells [55, 56]. The five *Trepomonas* lysozyme-family proteins are of at least two different origins (Fig. 3d, e). The first group has four members and multiple close homologs are found in the parabasalid *T. vaginalis* (Fig. 3d). These *Trepomonas* lysozyme-family proteins are likely of one or two bacterial origins because they are absent from other diplomonad genomes, although a common origin with the *Trichomonas* genes cannot be excluded. The second type is distantly related and found in a group of bacteria and a few sequences from fungi and water fleas (Fig. 3e). This lysozyme appears to have been acquired from bacteria by an ancestor of *S. vortens* and *Trepomonas* (Fig. 3e). A further adaptation to digest peptidoglycan constituents is suggested by the presence of a Glc*N*Ac kinase of bacterial origin (Additional file 5: Figure S3) that might allow *S. vortens* and *Trepomona*s to activate Glc*N*Ac for further utilization. Interestingly, activated Glc*N*Ac may shuttle into the pathway to synthesize *N*-acetylgalactosamine for building a cyst wall or into glucose metabolism.

In summary, the phylogenetic analyses support the hypothesis that *Trepomonas* recently acquired enhanced capabilities to degrade bacterial prey through gene transfers from bacteria (Figs. 2 and 3). There are no indications from the analyses that any of these genes have a eukaryotic ancestry, except that two of the genes are present in the parabasalid *T. vaginalis*. However, these genes are absent in all other sampled diplomonads. Thus, shared ancestry of these genes in *Trichomonas* and *Trepomonas* would imply three independent losses of each gene within diplomonads. Therefore, independent origins in *Trepomonas* and *Trichomonas* via gene transfer appear as a more likely scenario explaining the distribution of these two genes.

### Carbohydrate and amino acid metabolism

The carbohydrate and amino acid metabolism of *Trepomonas* sp. PC1 is similar to the predicted pathways of *S. salmonicida*, although *Trepomonas* has additional enzymes that have been acquired via lateral gene transfer (Table 2 and Additional file 2: Table S1). *Trepomonas* is predicted to be able to utilize glycerol as a carbon source due to possessing a laterally transferred glycerol dehydrogenase. It also seems to be able to utilize more carbon sources than the fish parasite *S. salmonicida* due to an extended set of 18 glycosyltransferases and glucosidases of uncertain specificity but of apparent bacterial origin. Furthermore, lateral transfers of α-amylase, α- and β-galactosidase as well as glucan endo-1,6-β-glucosidase contribute to an increased capacity to catabolize oligo- and polysaccharides. Together, this indicates an expansion of metabolic capacity to break down larger biomolecules to metabolites that feed into glycolysis.

### Extended nucleotide metabolism in *Trepomonas* sp. PC1

**Fig. 4:**
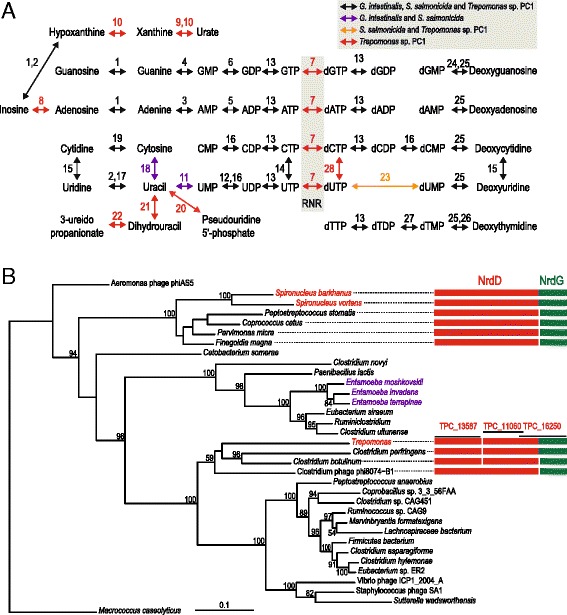
Nucleotide metabolism in diplomonads. **a** Proposed pathways for synthesis and scavenging of purine ribonucleosides, pyrimidine ribonucleosides and deoxynucleosides. The presence of enzymes across *G. intestinalis*, *S. salmonicida* and *Trepomonas* sp. PC1 is indicated by colors. Black: shared by all, purple: *G. intestinalis* + *S. salmonicida*, orange: *S. salmonicida* + *Trepomonas* sp. PC1, red: *Trepomonas* sp. PC1 only. Key to enzymes: 1. purine nucleoside phosphorylase, 2. inosine-uridine nucleoside N-ribohydrolase, 3. adenine phosphoribosyltransferase, 4. guanine phosphoribosyltransferase, 5. adenylate kinase, 6. guanylate kinase, 7. anaerobic ribonucleotide reductase (RNR), 8. adenosine deaminase, 9. xanthine dehydrogenase, 10. xanthine oxidase, 11. uracil phosphoribosyltransferase, 12. UMP kinase, 13. nucleoside diphosphate kinase, 14. CTP synthase, 15. cytidine deaminase, 16. UMP-CMP kinase, 17. uridine/thymine phosphorylase, 18. cytosine deaminase, 19. cytidine hydrolase, 20. pseudouridine-5’-phosphate glycosidase, 21. dihydrouracil dehydrogenase, 22. dihydropyrimidinase, 23. deoxyuridine-5’-triphosphate nucleotidohydrolase, 24. deoxyguanosine kinase, 25. deoxynucleosidase kinase, 26. thymidine kinase, 27. thymidylate kinase, and 28. deoxycytidine triphosphate deaminase. **b** Protein maximum likelihood phylogeny of class III anaerobic RNR of the NrdD class. The arrangement of NrdD and its activating protein NrdG in *Spironucleus*, *Trepomonas* and their closest relatives are indicated by boxes. Three *Trepomonas* transcripts make up a putative RNR. TPC1_13587 covers the N-terminal part of NrdD. TPC1_11060 and TPC1_16250 overlaps each other with 13 amino acids in the C-terminal part of NrdD. TPC1_16250 shows that NrdD is fused with NrdG in *Trepomonas*. The tree is inferred for NrdD, which is found in a single peptide in all species except *Trepomonas* and *Clostridium botulinum* and *Clostridium perfringens*. Eukaryotes are labeled according to their taxonomic/phylogenetic classification [13]: Amoebozoa (*purple*) and Excavata (*red*). Only bootstrap support values > 50 % are shown

A limited capacity for nucleotide metabolism is a common feature in host-associated organisms. Indeed, the human parasite *G. intestinalis* and the fish parasite *S. salmonicida* largely resort to scavenging purines and pyrimidines from their hosts [19, 20, 24], an option that is not available to a free-living organism such as *Trepomonas*. Interestingly, *Trepomonas* sp. PC1 has a more extensive nucleotide metabolism than *G. intestinalis* and *S. salmonicida* as a result of gene acquisitions (Table 2). Manual curation of the diplomonad nucleotide metabolic pathways identified 28 enzymes present in at least one of the three studied species (Fig. 4a). Just of these, 19 enzymes were present in both *G. intestinalis* and *S. salmonicida*, while all except two of the 28 are found within the *Trepomonas* transcriptome, and these could indeed still be present in the genome. *Trepomonas* encodes nine additional enzymes for nucleotide metabolism compared to *G. intestinalis*. Only one of these is present in the more closely related fish parasite *S. salmonicida*. Six out of the eight enzymes found only in *Trepomonas* have been acquired from bacterial sources (Fig. 4a and Additional file 2: Table S1). These additions make *Trepomonas* less dependent on scavenging. For example, inosine can be shuttled into purine metabolism by adenosine deaminase, and two enzymes, xanthine dehydrogenase and xanthine oxidase, may act to shuttle urate and xanthine into purine metabolism (Fig. 4a). In pyrimidine metabolism, *Trepomonas* may utilize 3-ureidopropionate and pseudouridine-5’-phosphate to generate uracil. The former pathway requires the concerted action of dihydropyrimidinase and dihydrouracil dehydrogenase and the latter is accomplished by pseudouridine-5’-phosphate glycosidase (Fig. 4a).

All life is dependent on a supply of deoxyribonucleotides as building blocks for DNA. Almost all organisms encode at least one RNR to catalyze the reduction of ribonucleotides to deoxyribonucleotides. The very few organisms that lack RNRs are all parasites or endosymbionts, including the amoebozoan *Entamoeba histolytica* and the diplomonads *G. intestinalis* and *S. salmonicida* [19–21]. This suggests that these parasitic diplomonads rely on deoxyribonucleotide scavenging and nucleoside kinases in the absence of a RNR [20, 24, 58]. *S. barkhanus* and *Spironucleus vortens* encode a class III anaerobic RNR of bacterial origin (Fig. 4b), indicating that they have re-gained the capability to reduce ribonucleotides. Three *Trepomonas* sp. PC1 transcripts together make a putative class III anaerobic RNR of the NrdD class, fused with the RNR-activating protein NrdG (Fig. 4b). Two considerations indicate that this fused gene has a bacterial origin distinct from *Spironucleus* RNR. First, the *Trepomonas* RNR is nested among bacterial sequences in the phylogenetic analysis, with a weak affinity to *Clostridium* and *Clostridium* phage sequences (Fig. 4b). Second, the *nrdD* and *nrdG* genes are frequently found in an operon in phages and bacteria [59]. The presence of RNR is predicted to make *Trepomonas* independent of scavenging of three of the four deoxynucleosides, however, the absence of thymidylate synthetase transcripts suggests that it may need a source of deoxythymidine (Fig. 4a).

### *Trepomonas* encodes a squalene-tetrahymanol cyclase (STC)

**Fig. 5:**
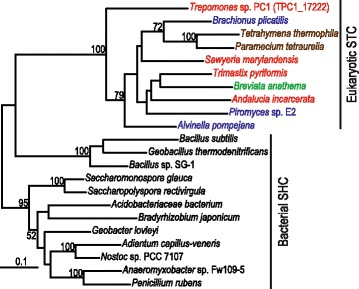
Phylogeny of eukaryotic STC and bacterial squalene-hopene cyclase (SHC). Protein maximum likelihood tree based on an alignment of the available eukaryotic STC sequences and representative bacterial SHC sequences. The tree is rooted based on a previous study that included oxidosqualene cyclase sequences [64]. Eukaryotes are labeled according to their classification [13]: Breviata (*green*), Excavata (*red*), Opisthokonta (*blue*), SAR (*brown*). Only bootstrap support values > 50 % are shown

Sterols are associated with fluidity and permeability of eukaryotic membranes and therefore are important for cellular processes such as phagocytosis [60, 61]. However, the oxygen-poor conditions where *Trepomonas* lives are not conducive to biosynthesis of sterols because this requires molecular oxygen [62]. There is recent evidence that several microbial eukaryotes that live under anoxic conditions employ the sterol substitute tetrahymanol, which can be synthesized without molecular oxygen [63, 64]. Tetrahymanol is present in the membrane of ciliates [32] and was recently demonstrated in the jakobid excavate *Andalucia incarcerata*, which is an anaerobe, but is not closely related to diplomonads [64]. We found that the *Trepomonas* transcriptome includes a homolog of STC, the enzyme required to synthesize tetrahymanol. This gene is absent from the genomes of *G. muris*, *G*. *intestinalis* and the three sampled *Spironucleus* species (*S. barkhanus*, *S. vortens* and *S. salmonicida*) as well as *T. vaginalis*, which is also an anaerobic parasite. These organisms may all acquire sterols from their eukaryotic hosts. By contrast, the presence of STC in *Trepomonas* could enable this diplomonad to feed solely on bacteria, and avoid the need to either access oxygen or scavenge sterols from a eukaryotic source. The *Trepomonas* protein branches with other eukaryotic STCs in the phylogeny, but without specific affinity to the STCs from other Excavata (Fig. 5). The pattern of STC presence and absence in eukaryotic genomes could either be explained by extensive gene-loss across eukaryotic branches (including three independent losses within diplomonads) or by eukaryote-to-eukaryote lateral gene transfers. The incompatibility of the STC phylogeny with the general eukaryote phylogenetic tree suggests at least some lateral gene transfer, but this could well be due to phylogenetic error in the STC tree, which has low taxon sampling and support values < 80 % for all relationships which suggest gene transfer events.

## Discussion

We have used a transcriptomics approach to examine the hypothesis that the free-living lifestyle of *Trepomonas* is a secondary adaptation. Our analyses show that this diplomonad encodes a large number of enzymes potentially involved in degradation of prey, including several enzyme families that degrade various parts of the bacterial cell walls and that are missing from the studied parasitic diplomonads (Fig. 2). Phylogenetic analyses showed that most prey-degradation enzymes were introduced recently into the *Trepomonas* genome via gene transfer, as expected if the organism is secondarily free-living (Fig. 3). The increased capacity of degradation of prey is complemented with a general increase of the metabolic capacity of *Trepomonas* compared to parasitic diplomonads, making it capable of utilizing more metabolites (Table 2 and Additional file 2: Table S1). This evolutionary path is indeed the reverse of what has been observed for the transition from a free-living to a parasitic lifestyle; the largest differences in the coding potential of the genome of the free-living *Bodo saltans* compared to parasitic kinetoplastids were shown to be within macromolecular degradation and catabolism of various metabolites from bacterial pray [5].

The increased metabolic capability in *Trepomonas* compared to parasitic diplomonads is especially striking for nucleotide metabolism, where the presence of nine additional enzymes, including an RNR, indicates that *Trepomonas* has adapted to a life less dependent on a host association than its ancestors (Fig. 4). In addition, we detected a homolog of the gene encoding STC, the enzyme required for the synthesis of tetrahymanol, a sterol surrogate [64]. This hallmark protein of free-living anaerobic phagotrophs possibly originated via eukaryote-to-eukaryote gene transfer (Fig. 5). We conclude that the transcriptome data favor the hypothesis that *Trepomonas* has adapted secondarily to a free-living lifestyle, over the alternative that the ancestral diplomonad was a free-living organism. This is supported by the organismal phylogeny where *Trepomonas* free-living diplomonads are nested within host-associated species (Fig. 1). However, there could exist free-living diplomonads within groups I–III, and a diplomonad normally found within a host could, in principle, have a cryptic free-living stage in its life cycle. To our knowledge, no such diplomonad has been observed. Only 10 diplomonad ribosomal RNA sequences from culture-independent surveys of environmental samples are present in the Silva ribosomal RNA gene database [65]. All of these belong to group IV (Additional file 6: Figure S4). This is in agreement with the view that group I–III diplomonads are parasites in the sense that they are taking resources from another organism, although they do not necessarily cause their host any negative effect.

The results challenge the assumption that parasitism is irreversible. Our data suggest that the adaptation to a free-living lifestyle occurred at least partly by acquisition of bacterial genes coding for prey-degrading enzymes needed by a free-living phagotroph. Such genes could have been acquired stepwise, and may have had a selective advantage also in the intestine of a host, if the diplomonad was able to ingest bacteria. Once the diplomonad has acquired some of the genes associated with a free-living lifestyle, it may be able to grow outside the host as trophozoites for short periods. The order of the events leading to adaptation to a free-living lifestyle in the ancestors of *Trepomonas* sp. PC1 could be deciphered by studying additional free-living diplomonads together with their closest host-associated relatives.

Adaptation by acquisition of prokaryotic genes is common in parasitic diplomonads [19, 45], suggesting that the ancestor of *Trepomonas* was exposed to bacterial genes. Such frequent lateral gene transfer probably was a precondition for the evolution of secondary free-living diplomonads. Lateral gene transfer has been proposed to be important in other parasites [66–69], which hints that evolution of secondary free-living taxa by gene acquisition could be a general phenomenon. There could, indeed, be examples of secondary free-living lineages in protist groups that include important human parasites such as *Entamoeba* (i.e., *Entamoebidae*) and *Trichomonas* (i.e., Parabasalia). Free-living lineages are nested within parasites in the phylogenetic trees of these groups [70–72], and lateral gene transfer has been shaping the metabolism of the parasites in the groups [66]. Interestingly, RNR, an essential enzyme for life independent of a host, has been lost in the human parasite *Entamoeba histolytica* [21], whereas we identified homologs of RNR of bacterial origins in three divergent *Entamoeba* species (Fig. 4b), including *E. terrapinae*, which is considered to be free-living [71]. This lineage might have adapted to a free-living lifestyle secondarily, similar to *Trepomonas*. If so, *E. terrapinae* is expected to harbor more recently acquired genes associated with a free-living lifestyle. This prediction could be tested by comparative studies of *Entamoeba* genomes.

Transitions from parasitic to free-living lifestyles might not be restricted to protist parasites. There are strong phylogenetic indications that it has happened at least once in the evolution of dust mites [10], and it has been suggested that the nematode genus *Rhabditophanes* is secondarily free-living [9]. Nematodes include both free-living taxa and members that are parasitic on animals or plants. The different lifestyles are admixed in the nematode phylogeny and it is estimated that parasitism has arisen at least 15 times independently [73]. Lateral gene transfer has contributed to parasitism in nematodes [67, 74], and it is reasonable to assume that it has also contributed to adaptation in free-living nematodes. Genomic studies are needed to understand the genetic basis and evolutionary history of the different lifestyles in this animal group.

## Conclusions

The argument against reversibility of parasitism is that it is improbable that an organism regains the same traits that were lost during the evolution to parasitism. Our study has shown that adaptation to a free-living lifestyle can occur via introduction of ‘foreign’ genes that the pre-parasitism free-living ancestor probably never encoded, thereby resolving the paradox. We believe that diplomonads and their closest relatives are a suitable group of organisms for future studies of evolutionary transitions between parasitic and free-living lifestyles.

## Methods

### Materials and sequencing

*Trepomonas* sp. PC1 was isolated from marine sediment near Peggy’s Cove, Nova Scotia, Canada, and grown in the lab on a mixed culture of bacteria. Total RNA was collected from several independently grown cultures in 50 mL Falcon tubes. Messenger RNA was purified from total RNA using the Poly(A)Purist™ MAG system (Ambion). Directed and size-fractionated cDNA libraries were made using the CloneMiner™ cDNA Library Construction Kit (Invitrogen), which were then sequenced with Sanger technology. The rest of the mRNAs were sequenced with Illumina Genome Analyzer IIx instrument, producing 41 million 100 bp long single reads. Raw RNA sequence reads were deposited at NCBI Sequence Read Archive (SRA) under accession number SRR2079337. The isolate is no longer in culture.

### Assembly

EST Sanger reads were assembled using Phred/Phrap v1.080812 [75]. The resulting contigs were manually examined and corrected in Consed v19.0 [76], which resulted in 1061 EST sequences.

The adapter sequence and the low quality bases at the ends of RNA-Seq reads affected the quality of the assembly, and were trimmed by cutadapt v2.6 [77] and prinseq v0.20.3 [78], respectively. Approximately 2 % of the low quality reads were removed and the remaining reads of high quality were assembled using Inchworm, the first part of Trinity [35]. Inchworm reconstructs full-length transcripts from RNA-Seq data without creating alternatively spliced isoforms; evidence from other sequenced diplomonads indicates that alternative splicing is a rare event [20]. Inchworm assembled 18,523 transcripts with size ≥ 200 nt. All EST sequences were found in the RNA-Seq assembly, except nine singleton EST reads that contribute no additional gene information. The annotations and analysis were thus only based on RNA-Seq data in this paper. Kmer abundance calculated by Inchworm was used to represent transcript expression.

### Annotation

Transcripts assembled from transcriptome data are often partial and the translating frame is not always the frame containing the longest open reading frame (ORF). BLASTX results could better capture the potential gene information in all six reading frames, however, BLASTX matches can become too fragmented to be significant. BLASTP using the longest ORF is a good complementary strategy to BLASTX when the longest ORF is the correct gene. In order to take advantage of both BLASTP and BLASTX results, we annotated the transcripts by combining the BLASTP results of the longest ORFs and the BLASTX results. We favor annotation from *S. salmonicida* because it is the closest sequenced relative with a manually annotated genome sequence [20]. The UniProtKB 20130905 database [79] and a database containing only *S. salmonicida* proteins were used for BLAST separately.

Genes that are positioned very close to each other or overlap each other can be assembled into a single transcript, since we do not have strand-specific or paired-end reads. Some transcript fusions could also result from incorrect assembly. To handle both cases, we first gathered all significant annotations belonging to the same transcript, allowing annotations to be partially or non-overlapping, and then systematically analyzed the reliability of an alternative translation. Manual efforts were also employed to better understand certain transcript fusions.

We annotated 7905 genes using this approach, with 42 transcripts coding for two genes. The longest ORFs were then searched against Pfam 25.0 [80] using HMMER3 [81], and 196 extra genes were added. For the rest of the transcripts that lack similarity to other genes or domains, we kept the longest ORFs with size ≥ 300 aa, which adds another 1879 genes, all of which contain in-frame TAA/TAG codons. In total, we had 9980 genes after the annotation step.

### Contamination removal

There were obvious cases of contamination among the annotated genes, which is not surprising as *Trepomonas* was grown with mixed bacteria. Contamination was first removed on the nucleotide sequence level. All the assembled transcripts were searched against the human genome and all the 2680 complete bacterial genomes available from NCBI (20131003) using BLASTN; 594 transcripts were determined to be from bacteria and 304 from human, using criteria that the e-value should be < 1 × 10^–10^ and > 50 % of the query sequence should be matched. Additional screening for contamination was then performed at the gene level. Since *Trepomonas* has TGA as its only stop codon, genes with in-frame TAA/TAG codons were likely to be true *Trepomonas* genes. A gene was excluded if it had no in-frame TAA/TAG codons, and its best BLASTP hit was to a bacterial sequence. This resulted in exclusion of 1605 likely bacterial genes. The two steps combined removed 1709 bacterial genes and 259 human genes, leaving 8012 *Trepomonas* genes.

All called genes with only a single in-frame TAA/TAG codon in the BLAST alignment and with best matches to prokaryotes were checked manually. The called gene was retained if the TAA/TAG coding for glutamine was in-frame and well within the BLAST alignment. If the TAA/TAG codon was in the beginning or the end of the BLAST alignment and there was no convincing alignment up- or downstream of the codon, the gene was removed from further analyses. In total, 61 genes were manually checked and 27 of them were removed. The numbers of in-frame TAA/TAG codons are reported for the genes detected as putative lateral gene transfers (Additional file 2: Table S1). In total, 7985 genes we retained for downstream analyses.

BUSCO [39] compares the input to its lineage-specific profile to quantitatively measure the completeness of the input in terms of the expected gene content. The lineage-specific profile “Eukaryota” containing 429 conserved eukaryotic proteins was downloaded from the BUSCO homepage, and setting “–m OGS” was used as gene set was used as the input.

This Transcriptome Shotgun Assembly project has been deposited at DDBJ/EMBL/GenBank under the accession GDID00000000. The version described in this paper is the first version, GDID01000000. Since TSA does not accept annotation on the complementary strand or an annotation that is internally partial, the transcript contigs were reverse-complemented for annotation on the complementary strand, were split into two if they coded for two genes, and were trimmed if they were internally partial.

### Protein families and orthologous groups

OrthoMCL v2.0.2 [82] was used to identify protein families in *Trepomonas*, as well as homologous groups shared by *Trepomonas* and *S. salmonicida*. E-value and match cutoff were set to be 1 × 10^–10^ and 40 %, respectively. OrthoMCL orthologous groups with only one member from each species were included in the protein identity analysis. Protein identities were extracted from the reciprocal BLAST results between *Trepomonas* and *S. salmonicida*.

### Analysis of pathways

The KEGG Automatic Annotation Server (KAAS) v1.69x using the bi-directional best hit (BBH) assignment method [83] was employed to predict pathways for each of *Trepomonas*, *S. salmonicida* and *G. intestinalis*, and the results were compared. The pathways reported in the main text were manually examined.

### Screening for lateral gene transfer candidates

PhyloGenie [47] and Darkhorse v1.5 [48] were combined to predict potential lateral gene transfer candidates. Both programs used the BLASTP results (against the nr database) of the predicted *Trepomonas* protein sequences. PhyloGenie constructs phylogenetic trees to search for gene transfer candidates. Darkhorse computes a lineage probability index to predict the potential gene transfer donors as well as recipients. This lineage probability index is supposed to be proportional to the phylogenetic distance between the database match sequence and the query sequence.

PhyloGenie uses HMMer v2.3.1 [84] to build a Hidden Markov Model (HMM) profile from the alignment in the BLAST results, and the full-length BLAST hits were used to search against the HMM profile to further select a subset to build the alignment. Neighbor-joining trees were first generated by mtrees provided in the package, and fed into Phylome Analysis Tool (PHAT), part of PhyloGenie, to pre-select the lateral gene transfer candidates.

RAxML v8.1.15 [85] was used to construct phylogenetic trees based on the alignments with the setting “-m PROTGAMMALG4X -f a -n 100” and PHAT was applied again to select trees of interest. Those trees were then manually inspected.

We selected trees with nodes that contain prokaryotes, diplomonads and up to four other eukaryotes, which selects the transfers from prokaryotes into diplomonads only, or into diplomonads, but shared with up to four other eukaryotes.

Phylogenie was run with the default parameter settings, except that we allowed sequences with maximum 70 % identity at the genus level (taxlevel = 1, maxsim = 0.7); and a maximum 90 % pairwise sequence similarity regardless of the species (maxhmmsim = 0.9). Further, we allowed up to 200 sequences in the alignment and 200 sequences to build the HMM profile.

During the manual inspection, donors and other eukaryotes in the same cluster were noted. Donor information at the genus level is also noted if there was a consistent genus and the bootstrap value was ≥ 70 %.

### Individual gene trees

The phylogenetic trees shown in the figures were generated as follows. Taxa were manually selected from the RAxML trees and the BLAST hits. Our draft genome sequences of *Giardia muris*, *Spironucleus barkhanus* and *Spironucleus vortens* were searched using tBLASTn, and identified homologs were added to the datasets. MAFFT v7.215 [86] with the L-INS-i option was used to align the sequences; BMGE v1.12 [87] with BLOSUM30 option was used to select sites to include in the phylogenetic analyses; RAxML was used with the same settings as described earlier to reconstruct the trees, except that the number of bootstrap replicates was increased to 500.

## Additional files

Additional file 1: Figure S1. Histogram of the protein identities. Based on 1692 1:1 orthologous pairs between *Trepomonas* sp. PC1 and *S. salmonicida*. Green line indicates the mean protein identity. (PDF 93 kb) Additional file 2: Table S1.
*Trepomonas* proteins identified as putative lateral gene transfers. (PDF 161 kb) Additional file 3: Phylogenetic trees of the genes identified as putative lateral gene transfers. All trees listed under heading tree# in Additional file 2: Table S1. (PDF 139 kb) Additional file 4: Figure S2. Protein maximum likelihood phylogeny of bactericidal permeability-increasing (BPI) proteins and BPI-like proteins. Eukaryotes are labeled according to their classification [13]: Amoebozoa (purple), Archaeplastida (green), Excavata (red) and Opisthokonta (blue). Only bootstrap support values > 50 are shown. (PDF 113 kb) Additional file 5: Figure S3. Protein maximum likelihood phylogeny of N-acetyl-D-glucosamine (GlcNAc) kinase. Eukaryotes are labeled according to their classification [13]: Amoebozoa (purple), Archaeplastida (green) and Excavata (red). Only bootstrap support values > 50 are shown. (PDF 258 kb) Additional file 6: Figure S4. Maximum likelihood phylogeny of 18S ribosomal RNA sequences. 18S sequences from species included in Fig. 1 and environmental sequences were downloaded from the Silva ribosomal RNA database. Taxa in green and black are isolated from environmental sources and host species, respectively. Only bootstrap support values > 50 are shown. (TRE 4366 kb)
