# Erratum to: On the reversibility of parasitism: adaptation to a free-living lifestyle via gene acquisitions in the diplomonad *Trepomonas* sp. PC1

**DOI:** 10.1186/s12915-016-0302-1

**Published:** 2016-09-12

**Authors:** Feifei Xu, Jon Jerlström-Hultqvist, Martin Kolisko, Alastair G. B. Simpson, Andrew J. Roger, Staffan G. Svärd, Jan O. Andersson

**Affiliations:** 1 Department of Cell and Molecular Biology, Science for Life Laboratory, Uppsala University, Uppsala, Sweden; 2 Department of Biology, Dalhousie University, Halifax, NS Canada; 3 Department of Biochemistry and Molecular Biology, Dalhousie University, Halifax, NS Canada; 4 Canadian Institute for Advanced Research, Integrated Microbial Biodiversity Program, Toronto, ON Canada; 5 Present address: Department of Medical Biochemistry and Microbiology, Uppsala University, Uppsala, Sweden; 6 Present address: Botany Department, University of British Columbia, Vancouver, BC Canada

## Erratum

Unfortunately, the original version of this article [1] contained an error. In the Discussion section, the species name *E. terrapinae* should be *E. moshkovskii* in two occasions. The corrected paragraph of the Discussion section can be found below;

“Interestingly, RNR, an essential enzyme for life independent of a host, has been lost in the human parasite *Entamoeba histolytica* [21], whereas we identified homologs of RNR of bacterial origins in three divergent *Entamoeba *species (Fig. 4b), including *E. moshkovskii*, which is considered to be free-living [71]. This lineage might have adapted to a free-living lifestyle secondarily, similar to *Trepomonas*. If so, *E. moshkovskii* is expected to harbour more recently acquired genes associated with a free- living lifestyle. This prediction could be tested by comparative studies of *Entamoeba *genomes.”
