# Supplementary material for: On the reversibility of parasitism: adaptation to a free-living lifestyle via gene acquisitions in the diplomonad Trepomonas sp. PC1
Source: BMC Biol. 2016 Aug 1;14:62. doi: 10.1186/s12915-016-0284-z (PMC4967989; doi:10.1186/s12915-016-0284-z)
Supplement: Additional file 1: Figure S1. — Histogram of the protein identities. Based on 1692 1:1 orthologous pairs between Trepomonas sp. PC1 and S. salmonicida. Green line indicates the mean protein identity. (PDF 93 kb) [file 12915_2016_284_MOESM1_ESM.pdf]

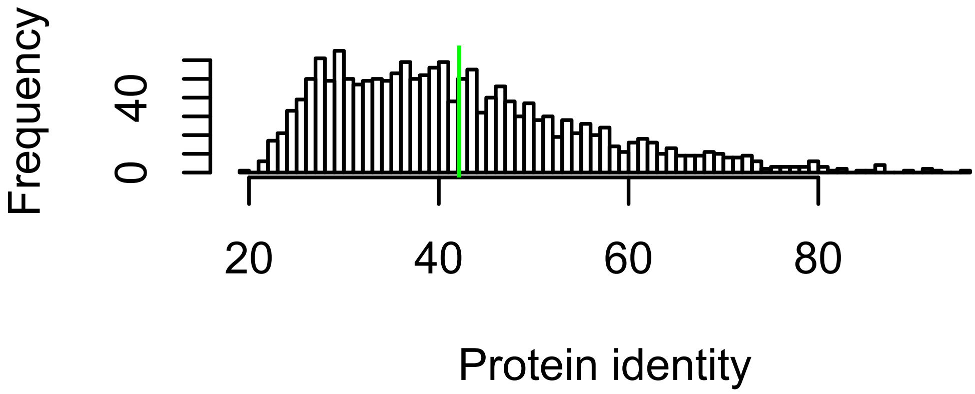

Figure S1. Histogram of the protein identities. Based on 1692 1:1 orthologous pairs between *Trepomonas* sp. PC1 and *S. salmonicida*. Green line indicates the mean protein identity.
