# Supplementary figures and images for: On the reversibility of parasitism: adaptation to a free-living lifestyle via gene acquisitions in the diplomonad Trepomonas sp. PC1
Source: BMC Biol. 2016 Aug 1;14:62. doi: 10.1186/s12915-016-0284-z (PMC4967989; doi:10.1186/s12915-016-0284-z)

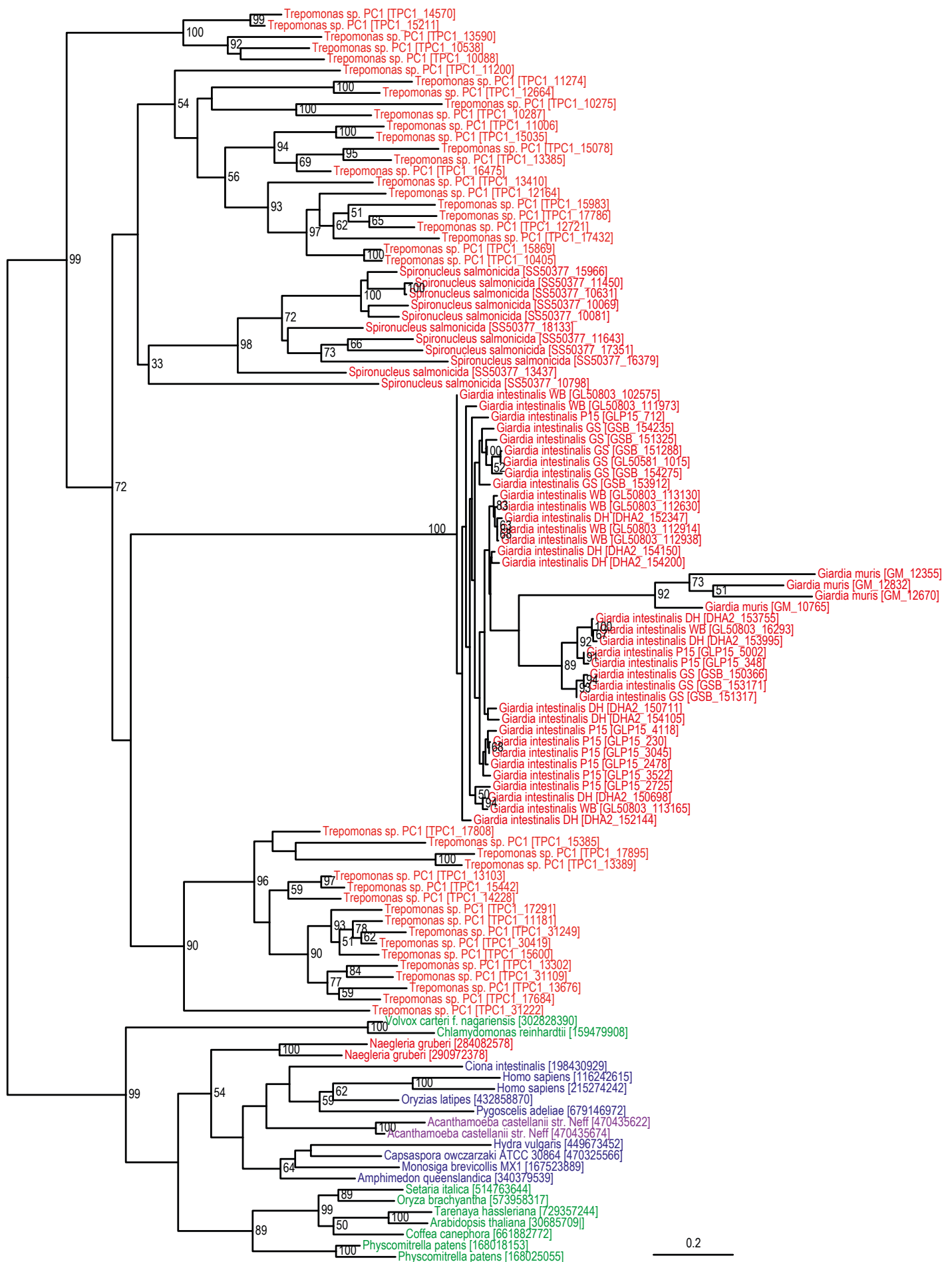

Supplement: Additional file 2: Table S1. — Trepomonas proteins identified as putative lateral gene transfers. (PDF 161 kb) [file 12915_2016_284_MOESM2_ESM.pdf]
