# Supplementary material for: On the reversibility of parasitism: adaptation to a free-living lifestyle via gene acquisitions in the diplomonad Trepomonas sp. PC1
Source: BMC Biol. 2016 Aug 1;14:62. doi: 10.1186/s12915-016-0284-z (PMC4967989; doi:10.1186/s12915-016-0284-z)
Supplement: Additional file 3: — Phylogenetic trees of the genes identified as putative lateral gene transfers. All trees listed under heading tree# in Additional file 2: Table S1. (PDF 139 kb) [file 12915_2016_284_MOESM3_ESM.pdf]

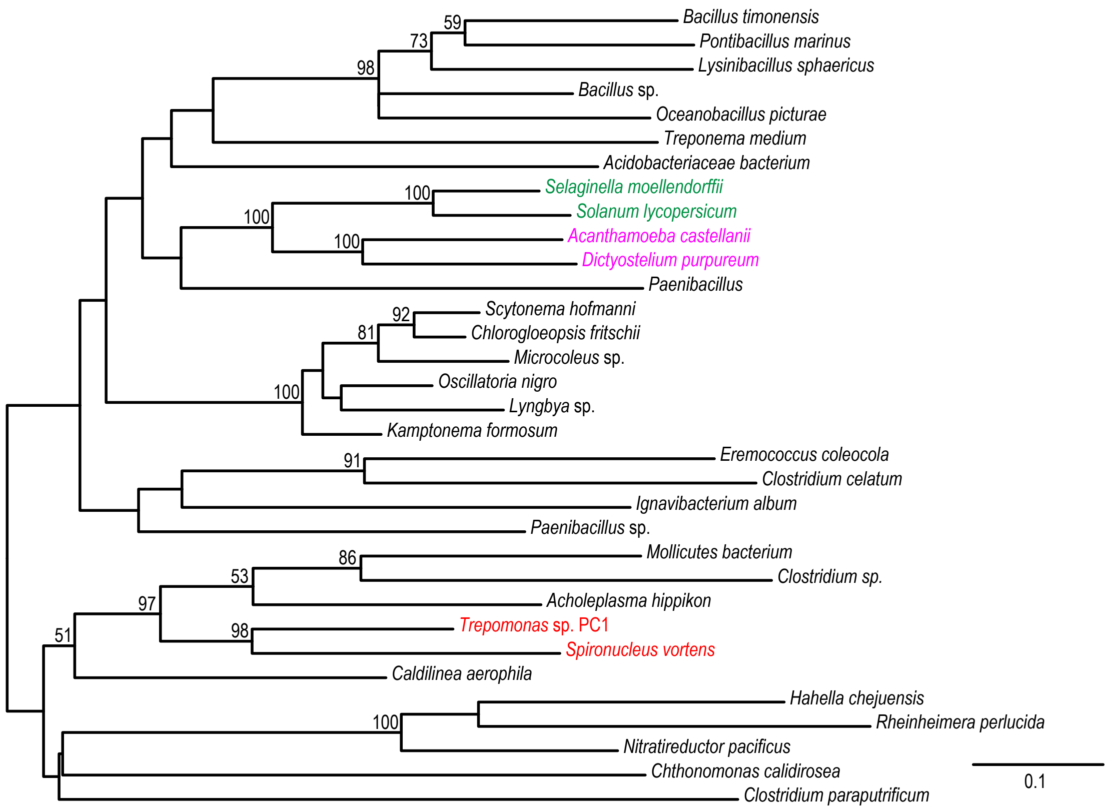

Figure S3. Protein maximum likelihood phylogeny of N-acetyl-D-glucosamine (GlcNAc) kinase. Eukaryotes are labeled according to their classification [2]: Amoebozoa (purple), Archaeplastida (green) and Excavata (red). Only bootstrap support values >50 are shown.
