# Supplementary material for: On the reversibility of parasitism: adaptation to a free-living lifestyle via gene acquisitions in the diplomonad Trepomonas sp. PC1
Source: BMC Biol. 2016 Aug 1;14:62. doi: 10.1186/s12915-016-0284-z (PMC4967989; doi:10.1186/s12915-016-0284-z)
Supplement: Additional file 4: Figure S2. — Protein maximum likelihood phylogeny of bactericidal permeability-increasing (BPI) proteins and BPI-like proteins. Eukaryotes are labeled according to their classification [13]: Amoebozoa (purple), Archaeplastida (green), Excavata (red) and Opisthokonta (blue). Only bootstrap support values > 50 are shown. (PDF 113 kb) [file 12915_2016_284_MOESM4_ESM.pdf]

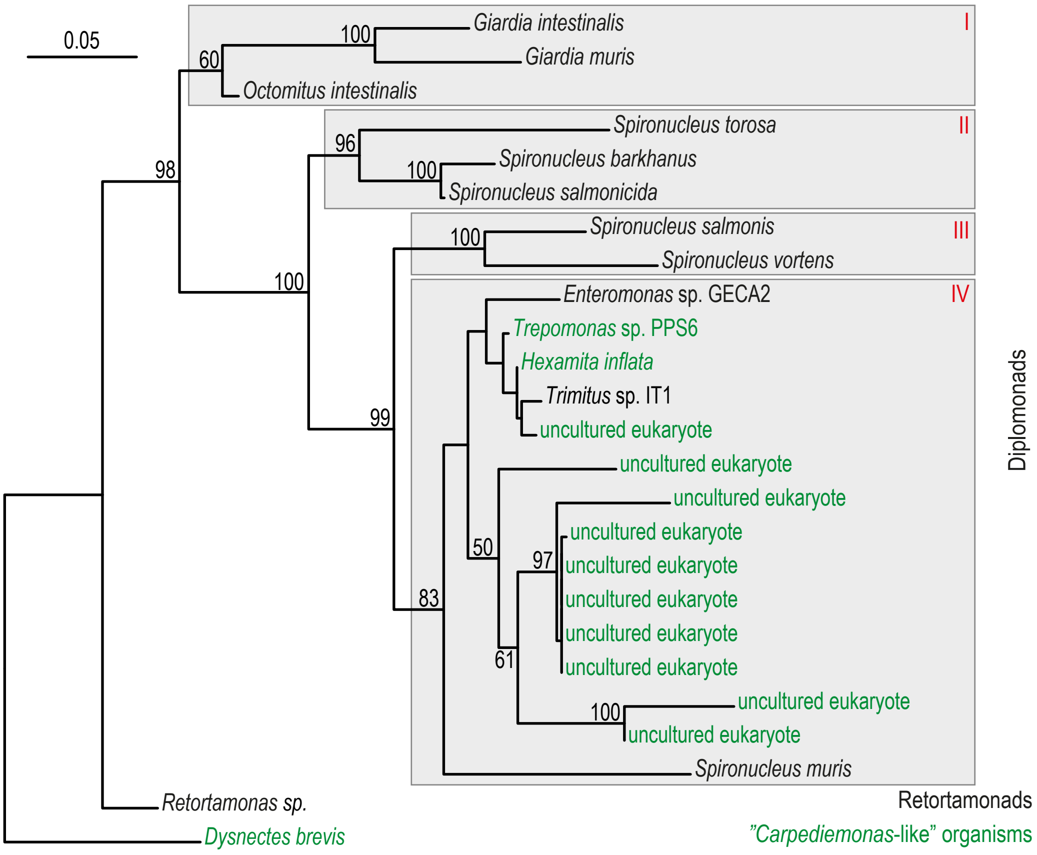

Figure S4. Maximum likelihood phylogeny of 18S ribosomal RNA sequences. 18S sequences from species included in Fig. 1 and environmental sequences were downloaded from the Silva ribosomal RNA database. Taxa in green and black are isolated from environmental sources and host species, respectively. Only bootstrap support values >50 are shown.
