# Supplementary material for: On the reversibility of parasitism: adaptation to a free-living lifestyle via gene acquisitions in the diplomonad Trepomonas sp. PC1
Source: BMC Biol. 2016 Aug 1;14:62. doi: 10.1186/s12915-016-0284-z (PMC4967989; doi:10.1186/s12915-016-0284-z)
Supplement: Additional file 5: Figure S3. — Protein maximum likelihood phylogeny of N-acetyl-D-glucosamine (GlcNAc) kinase. Eukaryotes are labeled according to their classification [13]: Amoebozoa (purple), Archaeplastida (green) and Excavata (red). Only bootstrap support values > 50 are shown. (PDF 258 kb) [file 12915_2016_284_MOESM5_ESM.pdf]

Table S1. *Trepomonas* proteins identified as putative lateral gene transfers.

Transcript	annotation	event#[a]	tree#[b]	#stops[c]	abundance[d]	Giardia[e]	Spiro[f]	other euks[g]	donor[h]	bootstrap[h]
TPC1_12469	[FeFe]-hydrogenase	217	163	6	2257	+	+	*Entamoeba*, *Retortamonas*	B	
TPC1_13661	[FeFe]-hydrogenase	217	163	8	248	+	+	*Entamoeba*, *Retortamonas*	B	
TPC1_14764	[FeFe]-hydrogenase	217	163	6	10	+	+	*Entamoeba*, *Retortamonas*	B	
TPC1_14871	2,3-bisphosphoglycerate-independent phosphoglycerate mutase	90	165	15	8	-	+		A	
TPC1_14500	2'-5' RNA ligase	214	153	5	4	-	-		B	
TPC1_15481	2'-5' RNA ligase	95	184	6	3	-	-		B	
TPC1_10119	4Fe-4S ferredoxin	144	5	1	209	-	-	*Entamoeba*	B	
TPC1_10418	4Fe-4S ferredoxin iron-sulfur binding domain protein	8	15	9	87	-	+		B	
TPC1_11134	4Fe-4S ferredoxin iron-sulfur binding domain protein	8	15	4	49	-	+		B	
TPC1_11650	4Fe-4S ferredoxin iron-sulfur binding domain protein	8	15	10	8	-	+		B	
TPC1_17692	4Fe-4S ferredoxin iron-sulfur binding domain protein	8	15	5	57	-	+		B	
TPC1_15879	4Fe-4S ferredoxin iron-sulfur binding domain-containing protein	104	203	2	92	-	-		B	
TPC1_12176	5'-methylthioadenosine nucleosidase, S-adenosylhomocysteine nucleosidase	41	75	5	27	+	+		B	
TPC1_10637	5'-nucleotidase SurE	36	69	2	8	-	-[j]		B	
TPC1_12058	5'-nucleotidase SurE	36	69	4	124	-	-[j]		B	
TPC1_11689	Acetyltransferase	32	56	10	4	-	-		B-Actinobacteria	95
TPC1_12740	Acetyl-transferase	50	99	12	5	-	-		B-Haloplasmatales	92
TPC1_11869	Acetyltransferase (GNAT) family protein	170	61	8	12	-	-		B	
TPC1_16988	Acyl-CoA synthetase	260	245	13	260	+	+	*Trichomonas*	B	
TPC1_17761	Acyl-CoA synthetase	260	245	11	804	+	+	*Trichomonas*	B	
TPC1_10594	Acyl-CoA--carboxylate coenzyme A transferase, family III	10	17	10	85	-	-		B	
TPC1_10685	Adenine phosphoribosyltransferase	14	23	4	19	+	+		B	

Transcript	annotation	event#[a]	tree#[b]	#stops[c]	abundance[d]	Giardia[e]	Spiro[f]	other euks[g]	donor[h]	bootstrap[h]
TPC1_14740	Adenosine deaminase	216	162	3	9	-	-		B	
TPC1_15380	Adenosine deaminase	216	162	7	76	-	-		B	
TPC1_11403	ADP-ribosylglycohydrolase	193	100	8	9	-	-	*Naegleria*, *Salpingoeca*	B	
TPC1_12772	ADP-ribosylglycohydrolase	193	100	11	13	-	-	*Naegleria*, *Salpingoeca*	B	
TPC1_12927	ADP-ribosylglycohydrolase	193	100	15	6	-	-	*Naegleria*, *Salpingoeca*	B	
TPC1_15113	ADP-ribosylglycohydrolase	193	100	5	74	-	-	*Naegleria*, *Salpingoeca*	B	
TPC1_15154	ADP-ribosylglycohydrolase	223	174	10	30	-	+		B	
TPC1_16786	ADP-ribosylglycohydrolase	223	174	8	158	-	+		B	
TPC1_17522	ADP-specific phosphofructokinase	139	259	8	81	-	+		A	
TPC1_11493	Alcohol dehydrogenase	29	52	6	29	-	+		B	
TPC1_10709	Alcohol dehydrogenase	35	66	4	2691	-	+		B	
TPC1_11970	Alcohol dehydrogenase	35	66	12	3	-	+		B	
TPC1_14538	Alcohol dehydrogenase	85	156	5	2858	-	+		B	
TPC1_15530	Alcohol dehydrogenase	96	187	5	75	-	+		B	
TPC1_16602	Alcohol dehydrogenase	119	230	15	15	+[k]	+[l]		B	
TPC1_16865	Alcohol dehydrogenase	126	240	0	5911	+	+		B	
TPC1_11677	Alcohol dehydrogenase E	167	55	2	15708	+	+		B	
TPC1_16833	Alcohol dehydrogenase E	257	238	2	1752	+	+		B	
TPC1_11144	Aldehyde dehydrogenase	21	41	27	57	-	+		B-Fusobacteria	82
TPC1_17087	Aldehyde dehydrogenase	21	41	4	45	-	+		B-Fusobacteria	82
TPC1_12708	Alpha amylase	192	98	9	8	-	-		B	
TPC1_12954	Alpha amylase	57	109	12	41	-	-		B	
TPC1_17202	Alpha amylase	57	109	8	443	-	-		B	
TPC1_12445	Alpha/beta hydrolase family protein	45	85	12	104	+	+		B	
TPC1_13796	Alpha/beta hydrolase family protein	45	85	6	60	+	+		B	
TPC1_14429	Alpha/beta hydrolase family protein	45	85	11	13	+	+		B	
TPC1_14454	Alpha/beta hydrolase family protein	45	85	3	3	+	+		B	
TPC1_12586	Alpha/beta hydrolase fold-containing	47	91	6	7	-	-		B-Proteobacteria	78

Transcript	annotation	event#[a]	tree#[b]	#stops[c]	abundance[d]	Giardia[e]	Spiro[f]	other euks[g]	donor[h]	bootstrap[h]
	protein									
TPC1_17405	Alpha/beta hydrolase fold-containing protein	114	220	11	26	-	-		B-Proteobacteria	84
TPC1_17868	Alpha/beta hydrolase fold-containing protein	114	220	9	13	-	-		B-Proteobacteria	84
TPC1_11670	Alpha/beta hydrolase fold-containing protein	270	220	6	6	-	-		B	
TPC1_16292	Alpha/beta hydrolase fold-containing protein	270	220	8	20	-	-		B	
TPC1_16708	Alpha/beta hydrolase fold-containing protein	121	232	2	5	-	-		B-Firmicutes	81
TPC1_15736	Alpha-galactosidase	100	195	8	30	-	-[j]		B	
TPC1_12584	Amidohydrolase family protein	187	90	8	102	-	-		B	
TPC1_15193	Amidohydrolase family protein	225	176	10	406	-	-	*Trichomonas*	B-Chlamydiae	80
TPC1_13069	Aminoacyl-histidine dipeptidase	199	118	8	80	+	+	*Blastocystis*	B	
TPC1_17722	Aminoacyl-histidine dipeptidase	199	118	3	4119	+	+	*Blastocystis*	B	
TPC1_16106	Aminoacyl-histidine dipeptidase	109	212	14	14	-	-[j]		B-Proteobacteria	99
TPC1_11535	Aminoglycoside phosphotransferase	30	53	10	3	-	-		B-Firmicutes	100
TPC1_14466	Aminotransferase	213	152	11	90	-	+		B	
TPC1_11060	Anaerobic ribonucleoside-triphosphate reductase	19	39	4	5	-	-		B	
TPC1_13587	Anaerobic ribonucleoside-triphosphate reductase	70	133	8	5	-	-		B	
TPC1_16250	Anaerobic ribonucleoside-triphosphate reductase	113	218	13	10	-	-		V	
TPC1_12015	ATPase, AAA family	176	68	5	3	-	+		B	
TPC1_10227	A-type flavoprotein	6	8	6	35	+	+		B	
TPC1_11949	A-type flavoprotein	6	64	8	5	+	+	*Trichomonas*	B	
TPC1_15207	A-type flavoprotein	6	64	7	151	+	+	*Trichomonas*	B	
TPC1_10917	A-type flavoprotein	6	128	18	33	+	+		B	
TPC1_13423	A-type flavoprotein	6	128	4	111	+	+		B	
TPC1_15158	A-type flavoprotein	6	128	6	562	+	+		B	
TPC1_16505	A-type flavoprotein	6	228	5	82	+	+	*Trichomonas*	B	
TPC1_12933	Beta-galactosidase	195	108	16	9	-	+	*Tetrahymena*	B	

Transcript	annotation	event#[a]	tree#[b]	#stops[c]	abundance[d]	Giardia[e]	Spiro[f]	other euks[g]	donor[h]	bootstrap[h]
TPC1_10343	Beta-lactamase superfamily domain-containing protein	152	20	9	31	-	-		B	
TPC1_10653	Beta-lactamase superfamily domain-containing protein	152	20	10	13	-	-		B	
TPC1_11041	Beta-lactamase superfamily domain-containing protein	152	20	6	4	-	-		B	
TPC1_14503	Beta-lactamase superfamily domain-containing protein	152	20	15	3	-	-		B	
TPC1_16221	Biotin operon repressor/biotin--[acetyl-CoA-carboxylase] synthetase	111	214	3	5	-[m]	-		B	
TPC1_11312	Calcineurin-like phosphoesterase	130	247	12	19	+	+		B	
TPC1_12598	Calcineurin-like phosphoesterase	130	247	14	6	+	+		B	
TPC1_13037	Calcineurin-like phosphoesterase	130	247	8	6	+	+		B	
TPC1_17158	Calcineurin-like phosphoesterase	130	247	8	12	+	+		B	
TPC1_12473	Carbamate kinase	184	86	4	397	+	+	*Carpediemonas*	B	
TPC1_15874	CDP-alcohol phosphatidyltransferase domain-containing protein	103	202	0	15	+	+		B-Proteobacteria	86
TPC1_15220	CDP-diacylglycerol-glycerol-3-phosphate 3-phosphatidyltransferase	226	177	2	53	+	+[l]		B	
TPC1_12914	Cell wall-associated hydrolase	56	107	2	1754	-	-		B-Bacteroidetes	72
TPC1_14960	Cell wall-associated hydrolase	219	166	1	242	-	-		B	
TPC1_11775	Cell wall-associated hydrolase, invasion-associated protein	3	3	4	17	-	-		B	
TPC1_12821	Cell wall-associated hydrolase, invasion-associated protein	3	3	6	16	-	-		B	
TPC1_14528	Cell wall-associated hydrolase, invasion-associated protein	3	3	4	56	-	-		B	
TPC1_10045	Cell wall-associated hydrolase, invasion-associated protein	231	182	2	26	-	-	*Trichomonas*	B	
TPC1_15346	Cell wall-associated hydrolase, invasion-associated protein	231	182	2	17	-	-	*Trichomonas*	B	
TPC1_16207	Cell wall-associated hydrolase, invasion-associated protein	231	182	6	42	-	-	*Trichomonas*	B	
TPC1_10785	Clan MH, family M20, peptidase T-like metallopeptidase	157	29	4	2712	-	-	*Trichomonas*	B	
TPC1_10503	Conserved hypothetical protein	9	16	10	11	-	-		B	
TPC1_12280	Conserved hypothetical protein	44	80	28	3	-	-		B	
TPC1_12567	Conserved hypothetical protein	186	89	28	1934	+	+		B	
TPC1_12830	Conserved hypothetical protein	52	103	2	1136	-	+		B	

Transcript	annotation	event#[a]	tree#[b]	#stops[c]	abundance[d]	Giardia[e]	Spiro[f]	other euks[g]	donor[h]	bootstrap[h]
TPC1_13765	Conserved hypothetical protein	72	136	5	4	-	-		B-Proteobacteria	98
TPC1_16724	Conserved hypothetical protein	72	136	1	9	-	-		B-Proteobacteria	98
TPC1_14603	Conserved hypothetical protein	88	159	2	2	-	-		B-Bacteroidetes	75
TPC1_12360	Conserved hypothetical protein	230	181	9	15	+	+		B	
TPC1_15334	Conserved hypothetical protein	230	181	8	45	+	+		B	
TPC1_15387	Conserved hypothetical protein	232	183	13	7	-	-		B	
TPC1_11905	Conserved hypothetical protein	133	251	7	5	-	+		B	
TPC1_14065	Conserved hypothetical protein	133	251	2	75	-	+		B	
TPC1_17297	Conserved hypothetical protein	133	251	0	13450	-	+		B	
TPC1_15085	CTP synthase	221	170	11	28	-	+		B	
TPC1_13600	Cyd operon protein YbgT	71	134	1	3	-	-		B	
TPC1_13324	Cysteine synthase A	205	126	3	4233	-	+		B	
TPC1_16598	Deoxycytidine triphosphate deaminase	254	229	9	19	-	-	*Dictyostelium*, *Entamoeba*, *Naegleria*	B	
TPC1_16233	Deoxyribose-phosphate aldolase	247	216	6	163	+	+		B	
TPC1_17796	Deoxyuridine 5'-triphosphate nucleotidohydrolase	267	266	1	50	-	-[j]		B	
TPC1_10071	Dihydropyrimidinase	39	73	17	272	-	-		A	
TPC1_12142	Dihydropyrimidinase	39	73	6	1776	-	-		A	
TPC1_10712	Dihydropyrimidine dehydrogenase	153	24	15	215	-	-		B	
TPC1_14840	DNA mismatch repair protein MutL	218	164	6	2	+	+	*Candida*, *Monosiga*	B	
TPC1_17450	DNA-binding protein, putative	137	257	1	2	-	-		B-Proteobacteria	82
TPC1_17303	DNA-directed RNA polymerase	135	253	5	18	-	+[l]		A	75
TPC1_12177	Ethanolamine ammonia-lyase large subunit (EutB) family protein	42	76	16	17	-	-		B	
TPC1_11309	Ethanolamine ammonia-lyase light chain (EutC) family protein	26	49	3	26	-	-		B	
TPC1_11509	Extracellular nuclease	190	96	12	15	+	+	*Trichomonas*	B	
TPC1_12647	Extracellular nuclease	190	96	2	209	+	+	*Trichomonas*	B	
TPC1_15484	Extracellular nuclease	265	264	5	175	+	+	*Trichomonas*	B	
TPC1_17709	Extracellular nuclease	265	264	5	247	+	+	*Trichomonas*	B	

Transcript	annotation	event#[a]	tree#[b]	#stops[c]	abundance[d]	Giardia[e]	Spiro[f]	other euks[g]	donor[h]	bootstrap[h]
TPC1_11248	FAD/FMN dependent oxidoreductase	25	46	3	2	-	+		B	
TPC1_16797	FAD/FMN dependent oxidoreductase	25	46	8	704	-	+		B	
TPC1_17518	FAD/FMN dependent oxidoreductase	25	46	10	18	-	+		B	
TPC1_12093	Fe-S protein assembly chaperone HscA domain-containing protein	37	70	3	3	-	+		B-Cyanobacteria	82
TPC1_15457	Fe-S protein assembly chaperone HscA domain-containing protein	37	70	24	9	-	+		B-Cyanobacteria	82
TPC1_16870	Fe-S protein assembly chaperone HscA domain-containing protein	37	70	6	5	-	+		B-Cyanobacteria	82
TPC1_17581	Fe-S protein assembly chaperone HscA domain-containing protein	37	70	19	25	-	+		B-Cyanobacteria	82
TPC1_13430	Formate C-acetyltransferase	67	129	10	476	-	-		B	
TPC1_14534	Fructokinase	83	154	17	5	+[k]	+[l]		B	
TPC1_16710	Fructose-1,6-bisphosphate aldolase	122	233	5	3801	+[k]	+[l]		B	
TPC1_10001	Glucan endo-1,6-beta-glucosidase	1	1	15	13	-	-		B	
TPC1_11777	Glucosamine-6-phosphate deaminase	169	59	14	13	+	+		B	
TPC1_12419	Glucose-6-phosphate isomerase	183	84	17	43	+	+	*Trichomonas*	B	
TPC1_11886	Glutamine synthetase	120	231	14	6	-	-		B-Firmicutes	74
TPC1_16673	Glutamine synthetase	120	231	4	6	-	-		B-Firmicutes	74
TPC1_10201	Glutamine synthetase catalytic region	145	6	11	6	-	-		B	
TPC1_14343	Glutamine synthetase type III	211	150	2	6	-	-		B	
TPC1_10233	Glutamine--fructose-6-phosphate aminotransferase [isomerizing]	146	9	16	118	-	-		B	
TPC1_10306	Glycerate kinase	149	13	14	23	-	+		B	
TPC1_13007	Glycerol dehydrogenase	198	116	13	82	-	-		B	
TPC1_10417	Glycosidase PH1107-related protein	132	249	8	42	-	-		B-Bacteroidetes	70
TPC1_17199	Glycosidase PH1107-related protein	132	249	13	9	-	-		B-Bacteroidetes	70
TPC1_10248	Glycoside hydrolase family 3 protein	147	10	11	700	-	-		B	
TPC1_15120	Glycosyl hydrolase family 2 protein	222	173	21	19	-	-		B	
TPC1_10339	Glycosyl transferase family 2 protein	31	14	6	3	-	-		B	
TPC1_14747	Glycosyl transferase family 2 protein	31	14	5	280	-	-		B	
TPC1_11552	Glycosyl transferase family 2 protein	31	54	3	6	-	-		B	

Transcript	annotation	event#[a]	tree#[b]	#stops[c]	abundance[d]	Giardia[e]	Spiro[f]	other euks[g]	donor[h]	bootstrap[h]
TPC1_15134	Glycosyl transferase family 2 protein	31	54	6	202	-	-		B	
TPC1_16989	Glycosyl transferase family 2 protein	31	54	6	466	-	-		B	
TPC1_17183	Glycosyl transferase family 2 protein	31	54	3	21	-	-		B	
TPC1_11304	Glycosyl transferases group 1 family protein	62	117	11	17	-	-		B	
TPC1_13016	Glycosyl transferases group 1 family protein	62	117	8	8	-	-		B	
TPC1_17473	Glycosyl transferases group 1 family protein	62	117	4	8	-	-		B	
TPC1_17626	Glycosyl transferases group 1 family protein	62	117	14	11	-	-		B	
TPC1_13228	Glycosyl transferases group 1 protein	202	123	9	5	-	-		B	
TPC1_12153	Glycosyltransferase	40	74	9	19	-	-		B	
TPC1_17062	Glycosyltransferase	138	258	9	4	-	-		B	
TPC1_17488	Glycosyltransferase	138	258	11	26	-	-		B	
TPC1_11246	GNAT family acetyltransferase	164	45	10	31	-	-		B	
TPC1_10729	HAD family hydrolase	15	25	9	11	-	-		B	
TPC1_12485	HAD family hydrolase	15	25	10	23	-	-		B	
TPC1_15952	HAD family hydrolase	15	25	12	8	-	-		B	
TPC1_11125	HAD family hydrolase	78	142	2	4	-	-		B	
TPC1_12218	HAD family hydrolase	78	142	2	7	-	-		B	
TPC1_14039	HAD family hydrolase	78	142	4	3	-	-		B	
TPC1_13754	Haloacid dehalogenase-like hydrolase domain-containing protein	78	142	6	65	-	-		B	
TPC1_17196	Haloacid dehalogenase-like hydrolase domain-containing protein	131	248	6	8	-	-		B	
TPC1_10675	Haloacid dehalogenase-like hydrolase family protein	13[n]	22	3	11	+	-		B	
TPC1_10898	Hemolysin III family protein	159	33	1	10	-	+		B-Thermotogae	71
TPC1_16467	Hemolysin III family protein	251	226	2	32	-	+[l]		B	
TPC1_14100	Histidinol phosphate phosphatase HisJ family protein	208	144	9	3	+	+[l]		B	
TPC1_16817	Histidinol phosphate phosphatase HisJ family protein	208	144	14	388	+	+[l]		B	
TPC1_11046	Homospermidine synthase	163	38	2	1749	-	-		B	
TPC1_16966	Hybrid cluster protein	259	244	7	378	+	+	*Entamoeba*	B	

Transcript	annotation	event#[a]	tree#[b]	#stops[c]	abundance[d]	Giardia[e]	Spiro[f]	other euks[g]	donor[h]	bootstrap[h]
TPC1_14995	Hydrolase, haloacid dehalogenase-like family	234	186	3	27	+	+		B	
TPC1_15505	Hydrolase, haloacid dehalogenase-like family	234	186	11	308	+	+		B	
TPC1_12607	Hydroxyacylglutathione hydrolase	188	92	11	10	+	+	*Salpingoeca*	B	
TPC1_15076	Hydroxyacylglutathione hydrolase	188	92	10	15	+	+	*Salpingoeca*	B	
TPC1_16889	Hydroxyacylglutathione hydrolase	188	92	5	30	+	+	*Salpingoeca*	B	
TPC1_17548	Hydroxyacylglutathione hydrolase	188	92	14	49	+	+	*Salpingoeca*	B	
TPC1_10673	Hypothetical protein	12	21	2	24	-	-		B-Proteobacteria	87
TPC1_11740	Hypothetical protein	33	58	5	550	-	-		B-Caldiserica	99
TPC1_12617	Hypothetical protein	49	94	4	2	-	-		B-Cyanobacteria	91
TPC1_13307	Hypothetical protein	203	124	9	330	+	+	*Trichomonas*	A-Euryarchaeota	100
TPC1_13657	Hypothetical protein	207	135	5	9	-	-		B	
TPC1_14116	Hypothetical protein	80	145	3	106	-	-		B	
TPC1_11116	Hypothetical protein	87	158	6	3	-	-		B	
TPC1_14559	Hypothetical protein	87	158	9	5	-	-		B	
TPC1_15016	Hypothetical protein	92	168	13	21	-	-		B	91
TPC1_10733	Hypothetical protein	99	194	8	352	-	-		B	
TPC1_11162	Hypothetical protein	99	194	5	10	-	-		B	
TPC1_15735	Hypothetical protein	99	194	8	45	-	-		B	
TPC1_17564	Hypothetical protein	99	194	9	45	-	-		B	
TPC1_17622	Hypothetical protein	99	194	4	173	-	-		B	
TPC1_16091	Hypothetical protein	108	211	7	37	-	-		B	
TPC1_11212	Inorganic diphosphatase	24	44	25	248	+	+		B	
TPC1_11681	Inorganic diphosphatase	24	44	16	10	+	+		B	
TPC1_11752	Inorganic diphosphatase	24	44	14	5	+	+		B	
TPC1_12030	Inorganic diphosphatase	24	44	25	57	+	+		B	
TPC1_13319	Inorganic diphosphatase	24	44	18	15	+	+		B	
TPC1_10032	Inosine-uridine nucleoside N-ribohydrolase	2	2	3	4	+	+		B-Proteobacteria	92
TPC1_12194	Inosine-uridine nucleoside N-ribohydrolase	2	2	4	6	+	+		B-Proteobacteria	92

Transcript	annotation	event#[a]	tree#[b]	#stops[c]	abundance[d]	Giardia[e]	Spiro[f]	other euks[g]	donor[h]	bootstrap[h]
TPC1_16010	Integral membrane protein	106	209	4	40	-	-		B	
TPC1_11014	Integrase core domain-containing protein	18	35	2	8	-	-		B-Aquificae	77
TPC1_17115	Integrase core domain-containing protein	18	35	5	25	-	-		B-Aquificae	77
TPC1_17442	Integrase core domain-containing protein	18	35	6	39	-	-		B-Aquificae	77
TPC1_11299	Integrase core domain-containing protein	166	48	2	3	-	-	*Trichomonas*	B	
TPC1_16923	Internalin	128	243	17	57	-	-		B	
TPC1_11947	ISPsy20, transposase IstB	172	63	1	3	-	-		B	
TPC1_17531	L-fucose isomerase, C-terminal domain-containing protein	264	260	13	18	-	-		B	
TPC1_15110	Lipolytic enzume, G-D-S-L family	94	172	8	55	+	+		B	
TPC1_15477	L-serine dehydratase	239	196	18	123	+	+	*Blastocystis*	B	
TPC1_15757	L-serine dehydratase	239	196	14	39	+	+	*Blastocystis*	B	
TPC1_15107	L-seryl-tRNA(Sec) kinase	93	171	6	4	-	+[l]		B	
TPC1_17861	Lysine-2,3-aminomutase	268	268	3	3	-	-		B	
TPC1_13093	LysM domain-containing protein	64	120	5	7	-	-		B-Firmicutes	94
TPC1_12384	Lysozyme family protein	182	83	2	6	-	-		B	
TPC1_14539	Lysozyme family protein	86	157	1	2858	-	-	*Trichomonas*	B	
TPC1_12700	Lysozyme family protein	271	157	4	192	-	-		B	
TPC1_13482	M18 family aminopeptidase	69	131	12	564	-	+[l]		B-Spirochaetes	83
TPC1_15939	M18 family aminopeptidase	105	205	11	560	-	+		B	
TPC1_10110	Major facilitator superfamily protein	11	19	11	67	+	+		B	
TPC1_10640	Major facilitator superfamily protein	11	19	13	18	+	+		B	
TPC1_10930	Major facilitator superfamily protein	160	34	4	28	+	+		B	
TPC1_12121	Major facilitator superfamily protein	160	34	3	5	+	+		B	
TPC1_11911	Major facilitator superfamily protein	38	71	11	36	+	+		B	
TPC1_12094	Major facilitator superfamily protein	38	71	5	12	+	+		B	
TPC1_12596	Major facilitator superfamily protein	38	71	9	12	+	+		B	
TPC1_13368	Major facilitator superfamily protein	38	71	7	29	+	+		B	
TPC1_14043	Major facilitator superfamily protein	38	71	3	17	+	+		B	
TPC1_16033	Major facilitator superfamily protein	38	71	9	187	+	+		B	

Transcript	annotation	event#[a]	tree#[b]	#stops[c]	abundance[d]	Giardia[e]	Spiro[f]	other euks[g]	donor[h]	bootstrap[h]
TPC1_17484	Major facilitator superfamily protein	38	71	6	5	+	+		B	
TPC1_10457	Major facilitator superfamily protein	77	141	9	32	+	+		B	
TPC1_13646	Major facilitator superfamily protein	77	141	9	10	+	+		B	
TPC1_13712	Major facilitator superfamily protein	77	141	13	55	+	+		B	
TPC1_13968	Major facilitator superfamily protein	77	141	9	7	+	+		B	
TPC1_15539	Major facilitator superfamily protein	77	141	10	11	+	+		B	
TPC1_15561	Major facilitator superfamily protein	77	141	5	4	+	+		B	
TPC1_16922	Major facilitator superfamily protein	77	141	7	7	+	+		B	
TPC1_15503	Major Facilitator Superfamily protein	233	185	1	11	-	-		B	
TPC1_12980	Mannose-6-phosphate isomerase	59	112	11	33	-	-		B	
TPC1_12364	MatE efflux family protein	181	82	5	23	+	+	*Trichomonas*	B	
TPC1_14906	MatE efflux family protein	181	82	1	48	+	+	*Trichomonas*	B	
TPC1_15610	MatE efflux family protein	125	190	19	347	+	+		B	
TPC1_11032	MatE efflux family protein	125	239	3	908	+	+		B	
TPC1_11668	MatE efflux family protein	125	239	4	111	+	+		B	
TPC1_15604	MatE efflux family protein	125	239	14	10	+	+		B	
TPC1_16836	MatE efflux family protein	125	239	10	22	+	+		B	
TPC1_17760	MatE efflux family protein	125	239	6	28	+	+		B	
TPC1_16712	MDR-type permease	123	234	11	83	+	+		B	
TPC1_12681	Metallo-beta-lactamase superfamily protein	191	97	6	602	+	+		B	
TPC1_16054	Metalloprotease, insulinase family protein	107	210	18	210	-	+		B-Proteobacteria	70
TPC1_16409	Metalloprotease, insulinase family protein	107	210	31	102	-	+		B-Proteobacteria	70
TPC1_13001	Methionine gamma-lyase	197	115	3	26	-	-		B	
TPC1_16747	Methionine gamma-lyase	124	236	4	252	-	-		B-Proteobacteria	75
TPC1_17410	Methionyl-tRNA synthetase	263	256	5	26	+	+		B	
TPC1_11697	Methylated-DNA--protein-cysteine methyltransferase	168	57	4	11	-	-		B	
TPC1_11946	Methyltransferase domain-containing protein	97	188	10	6	-	-		B-Chlamydiae	72
TPC1_15540	Methyltransferase domain-containing protein	97	188	3	5	-	-		B-Chlamydiae	72

Transcript	annotation	event#[a]	tree#[b]	#stops[c]	abundance[d]	Giardia[e]	Spiro[f]	other euks[g]	donor[h]	bootstrap[h]
TPC1_12998	Methyltransferase, FkbM family protein	209	148	5	4	-	-		B	
TPC1_14213	Methyltransferase, FkbM family protein	209	148	8	21	-	-		B	
TPC1_17489	Methyltransferase, FkbM family protein	209	148	8	43	-	-		B	
TPC1_12855	MFS permease (Drug)	54	105	7	49	-	-		B	
TPC1_12178	Mismatch repair protein	178	77	33	6	-[m]	+[l]	*Chaetomium, Myceliophthora*	B	
TPC1_12959	Molybdopterin biosynthesis MoeB protein	196	110	11	54	+	+[l]		B	
TPC1_13193	Myosin-cross-reactive antigen, putative	200	121	11	274	-	-	*Trichomonas*	B	
TPC1_13213	Myosin-cross-reactive antigen, putative	212	151	3	682	-	-	*Trichomonas*	B	
TPC1_14447	Myosin-cross-reactive antigen, putative	212	151	15	81	-	-	*Trichomonas*	B	
TPC1_14561	Myosin-cross-reactive antigen, putative	212	151	6	1370	-	-	*Trichomonas*	B	
TPC1_12843	N-acetyl-D-glucosamine kinase	272	270	1	30	-	-		B	
TPC1_10768	N-acetylmuramoyl-L-alanine amidase	156	28	3	55	-	-		B	
TPC1_11065	N-acetylmuramoyl-L-alanine amidase	156	28	5	14	-	-		B	
TPC1_11411	N-acetylmuramoyl-L-alanine amidase	156	28	9	34	-	-		B	
TPC1_16498	N-acetylmuramoyl-L-alanine amidase	156	28	3	922	-	-		B	
TPC1_11894	NADH oxidase	171	62	4	5	-[m]	+		B	
TPC1_12204	NADH oxidase	171	62	3	29	-[m]	+		B	
TPC1_12522	NADH oxidase	171	62	22	26	-[m]	+		B	
TPC1_14072	NADH oxidase	171	62	14	244	-[m]	+		B	
TPC1_17647	NADH oxidase	171	62	12	471	-[m]	+		B	
TPC1_13966	NADH oxidase	76	140	9	205	-[m]	+		B	
TPC1_14799	NADH oxidase	76	140	7	1379	-[m]	+		B	
TPC1_17869	NADH oxidoreductase	269	269	3	191	-	+		B	
TPC1_11192	NADPH oxidoreductase	22	42	3	1190	-[m]	+		B	
TPC1_10495	NADPH oxidoreductase	82	147	6	71	+	+		B	
TPC1_11001	NADPH oxidoreductase	82	147	6	52	+	+		B	
TPC1_14212	NADPH oxidoreductase	82	147	0	4677	+	+		B	
TPC1_13477	N-carbamyl-L-cysteine amidohydrolase	68	130	4	1738	-	-		B-Firmicutes	99
TPC1_12990	Nif3-related protein	61	114	5	35	+	+		B-Thermotogae	78

Transcript	annotation	event#[a]	tree#[b]	#stops[c]	abundance[d]	Giardia[e]	Spiro[f]	other euks[g]	donor[h]	bootstrap[h]
TPC1_15191	Nitrilase/cyanide hydratase and apolipoprotein N-acyltransferase	224	175	10	16	-	-		B	
TPC1_10295	Nitroreductase	148	12	17	32	+	+		B	
TPC1_14280	Nitroreductase	148	12	12	165	+	+		B	
TPC1_14902	Nitroreductase	148	12	9	761	+	+		B	
TPC1_16575	Nitroreductase	148	12	10	28	+	+		B	
TPC1_11030	Nitroreductase	161	36	2	3128	-	+		B	
TPC1_12813	Nitroreductase	51	101	1	14	-	-[j]		B	
TPC1_11240	NlpC/P60 family protein	194	102	3	98	-	-	*Trichomonas*	B	
TPC1_11430	NlpC/P60 family protein	194	102	7	19	-	-	*Trichomonas*	B	
TPC1_16273	NlpC/P60 family protein	194	102	2	20	-	-	*Trichomonas*	B	
TPC1_16368	NlpC/P60 family protein	194	102	4	53	-	-	*Trichomonas*	B	
TPC1_16504	NlpC/P60 family protein	194	102	4	332	-	-	*Trichomonas*	B	
TPC1_17596	NlpC/P60 family protein	194	102	5	4	-	-	*Trichomonas*	B	
TPC1_15239	NlpC/P60 family protein	227	178	3	49	-	-	*Trichomonas*	B	
TPC1_15794	NlpC/P60 family protein	227	178	2	38	-	-	*Trichomonas*	B	
TPC1_10609	NlpC/P60 family protein	231	182	5	55	-	-	*Trichomonas*	B	
TPC1_11218	NlpC/P60 family protein	231	182	2	168	-	-	*Trichomonas*	B	
TPC1_17359	NlpC/P60 family protein	231	182	5	117	-	-	*Trichomonas*	B	
TPC1_14527	NlpC/P60 family protein	115	221	3	26	-	-		B	
TPC1_10803	NMN adenylyl transferase and transcriptional regulator	158	30	10	5	-	-		B	
TPC1_16913	Ornithine transcarbamylase	258	242	3	2871	-	-[j]		B	
TPC1_14145	PD-(D/E)XK nuclease family transposase	81	146	6	4	-	-		B	
TPC1_17650	Peptidase T	142	263	1	2	-	+		B	
TPC1_16181	Peptidase T	266	265	9	40	-	+	*Entamoeba*	B	
TPC1_17778	Peptidase T	266	265	10	9	-	+	*Entamoeba*	B	
TPC1_16167	Peptide methionine sulfoxide reductase MsrA	110	213	10	243	+[k]	+		B	
TPC1_17381	Peptidoglycan-binding lysin domain containing protein	136	255	4	7	-	-		B-Firmicutes	84
TPC1_15291	Peptidyl-dipeptidase	228	179	11	806	-	+	*Entamoeba*	B	

Transcript	annotation	event#[a]	tree#[b]	#stops[c]	abundance[d]	Giardia[e]	Spiro[f]	other euks[g]	donor[h]	bootstrap[h]
TPC1_15012	Periplasmic solute-binding protein	91	167	1	2	-	-		B-Proteobacteria	98
TPC1_16423	Phosphoacetylglucosamine mutase	116	223	10	37	+	+		B	
TPC1_15786	Phosphoglycerate mutase	101	198	3	3	+	+		B	
TPC1_16733	Phosphoglycerate mutase	101	198	7	550	+	+		B	
TPC1_13403	Phosphotransferase family protein	65	127	8	2	-	-		B	
TPC1_14536	PP-loop superfamily protein	84	155	8	44	+	+		B	
TPC1_15699	Prolyl-tRNA synthetase	238	193	5	73	+	+[l]	*Trichomonas*	B	
TPC1_10098	Pseudouridine-5'-phosphate glycosidase	4	4	8	38	-	-		B	
TPC1_15967	Putative cysteine synthase	244	206	1	385	-	-		B	
TPC1_12115	Putative cytoplasmic protein	177	72	2	34	-	-	*Oxytricha*	B	
TPC1_12962	Putative glucoamylase	58	111	7	82	-	-		B	
TPC1_16446	Putative NADPH-flavin oxidoreductase	118	225	6	510	-	-		A	
TPC1_10236	Putative reverse transcriptase/endonuclease	140	261	30	110	+	+		B	
TPC1_10521	Putative reverse transcriptase/endonuclease	140	261	31	304	+	+		B	
TPC1_14372	Putative reverse transcriptase/endonuclease	140	261	8	84	+	+		B	
TPC1_15139	Putative reverse transcriptase/endonuclease	140	261	15	54	+	+		B	
TPC1_17609	Putative reverse transcriptase/endonuclease	140	261	20	19	+	+		B	
TPC1_12201	Putative selenium-dependent hydroxylase accessory protein YqeC	179	78	6	11	-	-		B	
TPC1_11094	Pyrazinamidase/nicotinamidase	20	40	5	158	+	+		B	
TPC1_15634	Pyridoxal-5'-phosphate-dependent protein beta subunit	237	191	6	2302	-	+[l]	*Trichomonas*	B	
TPC1_10745	Pyridoxal-phosphate dependent enzyme family protein	154	26	6	629	-	-	*Trichomonas*	B	
TPC1_12251	Pyrrolidone-carboxylate peptidase	201	122	4	8	-	-		B	
TPC1_13217	Pyrrolidone-carboxylate peptidase	201	122	4	2	-	-		B	
TPC1_11036	Pyruvate kinase	162	37	8	13	+	+		B	
TPC1_13574	Pyruvate-formate lyase-activating enzyme	206	132	3	15	+	-	*Blastocystis*	B	
TPC1_15796	Pyruvate-formate lyase-activating enzyme	206	199	10	77	+	-	*Trichomonas, Blastocystis*	B	

Transcript	annotation	event#[a]	tree#[b]	#stops[c]	abundance[d]	Giardia[e]	Spiro[f]	other euks[g]	donor[h]	bootstrap[h]
TPC1_12313	Radical SAM domain protein	180	81	6	4	-	-		B	
TPC1_15581	Radical SAM domain protein	235	189	12	4	-	-	*Dictyostelium, Rhizophagus*	B	
TPC1_15680	Redoxin domain protein	98	192	3	121	+	+		B	
TPC1_16239	Ribonuclease H	248	217	6	6	+	+	*Beauveria, Cordyceps, Trichosporon*	B	
TPC1_17210	Ribonuclease Z	261	250	3	2	-	-		B	
TPC1_17305	Ribose-phosphate pyrophosphokinase	262	254	4	184	+	+		B	
TPC1_12540	Ribosomal subunit interface protein, putative	46	87	2	5	-	-		B-Proteobacteria	98
TPC1_12777	Ricin superfamily protein	53	104	10	259	-	-		B	
TPC1_12838	Ricin superfamily protein	53	104	8	273	-	-		B	
TPC1_12870	Ricin superfamily protein	53	104	3	1267	-	-		B	
TPC1_12916	Ricin superfamily protein	53	104	9	351	-	-		B	
TPC1_11463	RT/endonuclease (Fragment)	140	261	25	4074	+	+		B	
TPC1_14434	RtcB protein	129	246	15	18	+	-		B	
TPC1_16537	RtcB protein	129	246	8	36	+	-		B	
TPC1_17111	RtcB protein	129	246	8	8	+	-		B	
TPC1_13878	RtcB protein	269	246	6	6	+	-		B	
TPC1_16224	Rubredoxin	112	215	2	2	-	-		B	
TPC1_15994	Rubrerythrin 1	245	207	3	2747	-	+		B	
TPC1_16884	Selenide, water dikinase	127	241	16	278	-	+		B	
TPC1_12988	Serine acetyltransferase	60	113	2	181	-	+		B	
TPC1_15718	Serine acetyltransferase	60	113	4	57	-	+		B	
TPC1_15766	SPFH domain/Band 7 family protein	240	197	4	919	-	-	*Ichthyophthirius, Tetrahymena*	B	
TPC1_14612	Streptococcal 67 kDa myosin-cross-reactive antigen like family protein	215	160	1	183	-	-	*Trichomonas*	B	
TPC1_15304	Streptococcal 67 kDa myosin-cross-reactive antigen like family protein	229	180	2	897	-	-	*Trichomonas*	B	
TPC1_13903	Structural maintenance of chromosomes protein	74	138	46	32	-[m]	-[j]		B-Firmicutes	72
TPC1_15922	Succinyl-CoA ligase like flavodoxin domain-containing protein	243	204	3	52	-	+		B	

Transcript	annotation	event#[a]	tree#[b]	#stops[c]	abundance[d]	Giardia[e]	Spiro[f]	other euks[g]	donor[h]	bootstrap[h]
TPC1_13859	Sugar kinase	73	137	14	73	-	-		B	
TPC1_11794	Sugar phosphate isomerase	34	60	12	287	-	-		B-Firmicutes	74
TPC1_10839	Sulfide dehydrogenase	17	32	14	675	+	+		B	
TPC1_12608	Superoxide reductase	48	93	0	5017	+[k]	+[l]		A	
TPC1_15995	Tetratricopeptide repeat protein	246	208	43	21	+	+		B	
TPC1_12556	Thermophilic metalloprotease (M29) family protein	185	88	12	142	-	+		B	
TPC1_16260	Thermophilic metalloprotease (M29) family protein	185	88	12	27	-	+		B	
TPC1_13737	Thioredoxin domain-containing protein	117	224	4	38	+	+		B	
TPC1_16442	Thioredoxin domain-containing protein	117	224	1	6948	+	+		B	
TPC1_11291	Thiosulfate sulfurtransferase	165	47	13	80	+	+		B	
TPC1_15867	Threonine synthase	242	201	5	64	-[m]	-[j]	*Trichomonas*	B	
TPC1_17300	Transcription activator effector binding	134	252	1	233	-	-		B	
TPC1_10808	Transglutaminase domain protein	16	31	6	4	-	-		B-Verrucomicrobia	100
TPC1_17366	Transglutaminase domain protein	16	31	10	8	-	-		B-Verrucomicrobia	100
TPC1_11449	Transglutaminase-like superfamily protein	27	50	10	5	+	+		B	
TPC1_11584	Transglutaminase-like superfamily protein	27	50	3	7	+	+		B	
TPC1_11691	Transglutaminase-like superfamily protein	27	50	10	134	+	+		B	
TPC1_15656	Transglutaminase-like superfamily protein	27	50	9	163	+	+		B	
TPC1_16279	Transglutaminase-like superfamily protein	27	50	13	24	+	+		B	
TPC1_17799	Transglutaminase-like superfamily protein	27	50	6	31	+	+		B	
TPC1_10101	Transglutaminase-like superfamily protein	75	139	13	37	+[k]	+[l]		B	
TPC1_11836	Transglutaminase-like superfamily protein	75	139	4	25	+[k]	+[l]		B	
TPC1_13112	Transglutaminase-like superfamily protein	75	139	5	17	+[k]	+[l]		B	
TPC1_13963	Transglutaminase-like superfamily protein	75	139	3	11	+[k]	+[l]		B	
TPC1_14925	Transglutaminase-like superfamily protein	75	139	18	65	+[k]	+[l]		B	
TPC1_17743	Transglutaminase-like superfamily protein	75	139	16	36	+[k]	+[l]		B	
TPC1_10215	Transmembrane domain-containing protein	5	7	10	3	-	-		B	
TPC1_13082	Transmembrane domain-containing protein	63	119	7	385	-	-		B	

Transcript	annotation	event#[a]	tree#[b]	#stops[c]	abundance[d]	Giardia[e]	Spiro[f]	other euks[g]	donor[h]	bootstrap[h]
TPC1_14676	Transmembrane domain-containing protein	89	161	1	113	-	-		B-Actinobacteria	77
TPC1_11482	Transposase	28	51	3	3	-	-		B	
TPC1_15359	tRNA (5-methylaminomethyl-2-thiouridylate)-methyltransferase	143	267	5	21	-	+		B-Fusobacteria	97
TPC1_17832	tRNA (5-methylaminomethyl-2-thiouridylate)-methyltransferase	143	267	16	180	-	+		B-Fusobacteria	97
TPC1_11987	UDP-glucose 4-epimerase	175	67	4	74	+	+	*Albugo*, *Phytophthora*, *Thalassiosira*	B	
TPC1_14059	UDP-glucose 4-epimerase	79	143	7	15	-	+		B	
TPC1_13309	Uracil-DNA glycosylase	204	125	5	12	+	+	*Naegleria*	B	
TPC1_12817	Uridine kinase	256	149	11	9	+	+	*Entamoeba*	B	
TPC1_14335	Uridine kinase	256	149	3	51	+	+	*Entamoeba*	B	
TPC1_13475	Uridine kinase	256	237	12	440	+	+	*Entamoeba*, *Naegleria*	B-Spirochaetes	91
TPC1_16769	Uridine kinase	256	237	11	230	+	+	*Entamoeba*, *Naegleria*	B-Spirochaetes	91
TPC1_10095	von Willebrand factor type A domain-containing protein	189	95	8	120	-	-		B	
TPC1_12619	von Willebrand factor type A domain-containing protein	189	95	1	76	-	-		B	
TPC1_15206	von Willebrand factor type A domain-containing protein	189	95	12	426	-	-		B	
TPC1_17840	von Willebrand factor type A domain-containing protein	189	95	9	53	-	-		B	
TPC1_10764	von Willebrand factor type A domain-containing protein	189	219	3	126	-	-		B	
TPC1_16258	von Willebrand factor type A domain-containing protein	189	219	2	177	-	-		B	
TPC1_11208	Xaa-Pro dipeptidase	23	43	4	117	+[k]	+		B	
TPC1_12876	Xanthine dehydrogenase accessory factor	55	106	11	151	-	-		B	
TPC1_10271	YqeB family selenium-dependent molybdenum hydroxylase system protein	7	11	9	96	-	-		B	

[a]) Each number refer to a inferred gene transfer event.

[b]) Number refer to the reference tree in S1 File.

[c]) Number of in-frame TAA or TAG codons in the inferred *Trepomonas* open reading frame within the BLAST alignment against the best database match.

[d]) Transcript expression as represented by Kmer abundance calculated by Inchworm.

[e]) Presence of absence of a homolog with the same origin in *G. intestinalis*.

[f]) Presence of absence of a homolog with the same origin in *S. salmonicida*.

[g]) Indicate if sequences from non-diplomonad eukaryotes are branching with diplomonas in the tree.

[h]) Inferred donor lineage. A indicates Archaea and B Bacteria.

[i]) Bootstrap support value for the identification of specific donor group.

[j]) *S. salmonicida* encodes a distantly related homolog

[k]) G. intestinalis encodes a homolog not included in the tree

[l]) *S. salmonicida* encodes a homolog not included in the tree

[m]) *G. intestinalis* encodes a distantly related homolog

[n]) Two transfers, one into *Giardia* and one into *Trepomonas*
